# The Chemistry and Biology of the Tetrodotoxin Natural Product Family

**DOI:** 10.1002/anie.202502404

**Published:** 2025-08-01

**Authors:** Benedikt Nißl, Marcel Mülbaier, Francesca Grisoni, Dirk Trauner, Marcel Bermúdez, Clemens Dialer, David B. Konrad

**Affiliations:** ^1^ Department of Pharmacy Ludwig‐Maximilians‐Universität München Butenandtstr. 5–13, Haus C 81377 Munich Germany; ^2^ Drug Discovery Engine Grünenthal GmbH Zieglerstr. 6 52078 Aachen Germany; ^3^ Biomedical Engineering Department Eindhoven University of Technology Groene Loper 3 Eindhoven The Netherlands; ^4^ Department of Chemistry University of Pennsylvania 231 South 34th Street Philadelphia PA 19104‐6323 USA; ^5^ Institute of Pharmaceutical and Medicinal Chemistry University of Münster Corrensstr. 48 48149 Münster Germany; ^6^ Department of Pharmaceutical Sciences University of Vienna Josef‐Holaubek‐Platz 2 Vienna 1090 Austria

**Keywords:** Chemical probes, Medicinal chemistry, Nociception, Total synthesis, Voltage‐gated sodium channels

## Abstract

Tetrodotoxin is a neurotoxic marine alkaloid, first isolated in 1909 from pufferfish and named after the biological order tetraodontiformes. Since its structural elucidation in 1964, it has attracted the interest of synthetic organic chemists due to its exceptional polarity, complex architecture, and important biological activity. This review highlights the diversity of the tetrodotoxin natural product family and discusses the origins of derivatives, biosynthetic hypotheses, and biological activities. Furthermore, potential therapeutic applications and structure–activity relationship studies are covered, along with the total syntheses of the natural product and selected derivatives that were published to date.

## Graphical abstract

Tetrodotoxin is best known as the poison of the pufferfish, which is a delicacy in Japanese cuisine. Its toxicity arises from the selective blockage of Na_V_ channels, which is depicted for the Na_V_1.7 pore, highlighting the DEKA motif, which permeates sodium ions. Depending on the subtype, inhibiting Na_V_s can lead to respiratory paralysis (Na_V_1.4) and heart failure (Na_V_1.5) or promote the treatment of pain (Na_V_1.7‐Na_V_1.9).

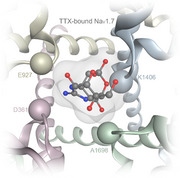

## 1 Introduction

**Figure 1:**
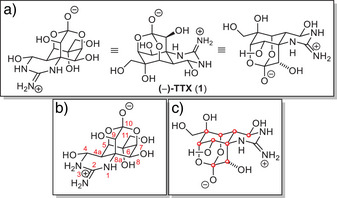
a) Different representations of TTX. b) Systematic numbering of the carbon skeleton. c) Highlighted stereocenters.

Tetrodotoxin (TTX, **1**) is best known as the poison of the Fugu pufferfish, which can be deadly when wrongly prepared and consumed as a delicacy (Figure 1). For humans, TTX is one of the most lethal non‐proteinaceous toxins with a low molecular weight.^[^ ^1^ ^]^ It selectively blocks voltage‐gated sodium channels (VGSC or Na_V_), which are involved in the transmission of electric signals between the body and the brain. TTX has a preference for Na_V_1.1–1.4 and Na_V_1.6–1.7,^[^ ^2^ ^]^ which inhibits the formation and propagation of action potentials, leading to paralysis, respiratory failure, and, without a timely medical intervention, to death.

The name tetrodotoxin is derived from tetraodontiformes, an order that includes pufferfish. It was introduced by Tahara who first isolated TTX in 1911.^[^ ^3^ ^]^ However, the isolation of a pure, crystalline sample by Yokoo was achieved only four decades later.^[^ ^4^ ^]^ The molecular structure was elucidated through the independent works of Woodward,^[^ ^5^ ^]^ Goto,^[^ ^6^ ^]^ and Tsuda,^[^ ^7^ ^]^ in 1964 and subsequently confirmed by X‐ray crystallography.^[^ ^8^ ^]^ Remarkably, Kishi and coworkers published the first synthetic route toward this highly unusual natural product shortly thereafter in 1972.^[^ ^9^, ^10^, ^11^, ^12^ ^]^ Over the past five decades, many other synthetic studies have been reported.^[^ ^13^, ^14^ ^]^ The continued fascination with TTX as a synthetic target and as a chemical probe, the recently published insights into its mode of action, as well as the identification of a plethora of derivatives and new hypotheses regarding its biosynthesis have prompted us to write this review.

## 2 Interaction of Tetrodotoxin with Voltage‐Gated Sodium Channels

### 2.1 Composition and Sodium Permeability of VGSCs

**Figure 2:**
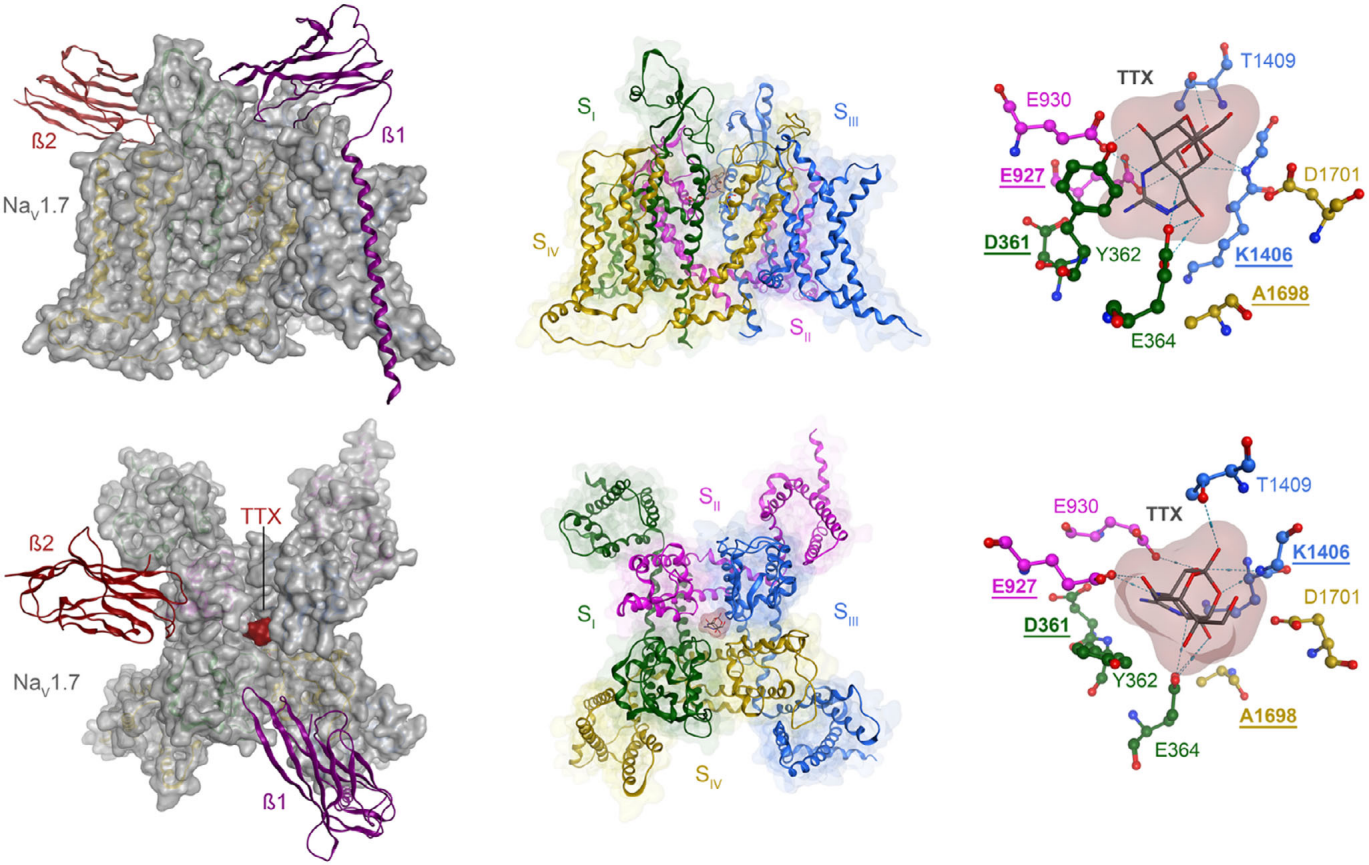
Structural overview of Na_V_1.7 in complex with TTX (**1**) and auxiliary β‐subunits (PDB entry 6J8I). On the left, the whole complex is shown: the α‐subunit (grey surface), along with the auxiliary β‐subunits (ribbons). The organization of the α‐subunit into four segments is illustrated in the middle. Each segment consists of a voltage‐sensing domain and a pore‐forming domain, where the latter is located in the center of the α‐subunit. On the right, the binding site of TTX (**1**) is shown with key residues for ligand binding. The residues of the DEKA motif are in bold and underlined. The top depiction highlights the transmembrane view, and the bottom depiction highlights the extracellular view.

Voltage‐gated sodium channels (VGSCs) are multi‐subunit transmembrane proteins that are activated by voltage changes in the cell membrane and consist of nine different subtypes in humans, which are termed Na_V_1.1–Na_V_1.9. Structurally, they are composed of one large α‐subunit (220–260 kDa), and one or more smaller auxiliary β‐subunit(s) (33–36 kDa). The α subunit is composed of four segments (DI–DIV), each containing a voltage‐sensing domain (VSD1–4), and a pore‐forming domain (Figure 2) that allows for the selective flux of sodium ions into the cell. VGSCs are structurally related to homotetrameric voltage‐gated potassium channels (VGKC or K_V_), which suggests that VGSCs might have evolved from early potassium channels.^[^ ^15^, ^16^ ^]^

To selectively conduct either sodium or potassium ions, both channel families rely on alternate mechanisms, which are mediated by their selectivity filters (SF).^[^ ^17^, ^18^ ^]^ In VGKC channels, it comprises a TVGYG amino acid sequence, which mimics the aqueous solvation shell of K^+^ through its coordination with carbonyl oxygens.^[^ ^19^ ^]^ In studies of cloned mammalian VGSCs, the four crucial residues aspartate–glutamate—lysine–alanine (DEKA) within the selectivity filter region were identified.^[^ ^20^, ^21^, ^22^, ^23^ ^]^ Selective passage of sodium ions through the pore is facilitated by the inclusion of a lysine side chain into this selectivity filter, which forms a network of hydrogen bonds with the adjacent aspartate and glutamate carboxylates, as well as the alanine carbonyl group. These interactions result in the constriction and stabilization of the pore, thereby enabling the specific binding of sodium over larger potassium ions. Mutating DEKA to EEEE, which corresponds to the selectivity filter in voltage‐gated calcium channels (VGCCs) transforms the human Na_V_ channel into a Ca^2+^ selective channel.^[^ ^24^, ^25^ ^]^

**Figure 3:**
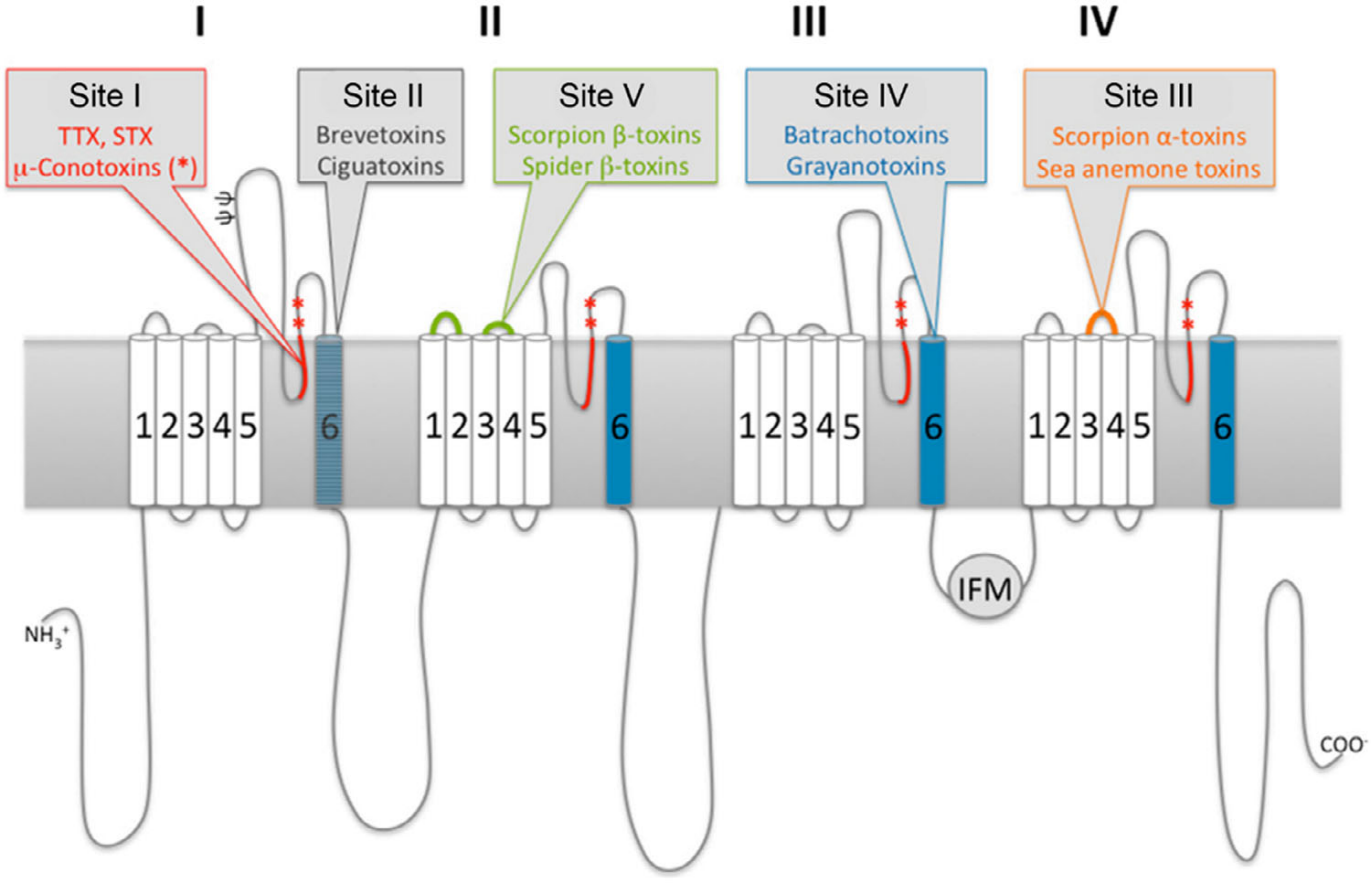
Two‐dimensional representation^[^ ^28^ ^]^ of the large α‐subunit of VGSC indicating the position of the five located neurotoxin binding sites. TTX (**1**) is bound between the four domains (red highlighted loops) within the membrane, blocking the pore and preventing sodium ion passage. Copyright: © 2011 Stevens, Peigneur, and Tytgat (the numbering from the different sites was changed from Roman to Greek numbers).^[^ ^28^ ^]^

Multiple binding sites are available for VGSC inhibitors, six of which can be targeted by neurotoxins (Site I to Site VI, with only five being well‐defined and characterized).^[^ ^26^, ^27^, ^28^ ^]^ Such binding sites differentiate by the mode of action, the specific domain, and the resulting effect (Figure 3).^[^ ^17^ ^]^ Neurotoxins can interfere with VGSCs in two different ways: by blocking the pore (thus preventing sodium ion passage) or, with higher prevalence, by modifying the gating mechanism. The central cavity formed by the α‐subunit on the extracellular surface is termed Site I. This site is targeted by small‐molecule guanidinium toxins like TTX and saxitoxin (STX) as well as short peptides like μ‐conotoxins.^[^ ^28^ ^]^ Site II, located on top of segment 6 between domains III and IV, binds to small lipid‐soluble alkaloid toxins such as batrachotoxin and veratridine.^[^ ^29^ ^]^ Site III and Site IV both consist of extracellular loops in VSDII and VSDIV, respectively, and are targeted by small peptide toxins from scorpions, anemones, and spiders like huwentoxins (HWTX‐II), prototoxin‐II, and tityus gamma toxin, which were investigated as pharmacological targets by various companies and groups for their potential to treat pain.^[^ ^30^ ^]^ Janssen Pharmaceuticals, for instance, developed analogues of the short peptide ProTX‐II binding to Site IV within the VSDII.^[^ ^31^ ^]^ Site V, which is formed by a bridge between domains I and IV, is targeted by ladder polyethers, such as brevetoxin 1.^[^ ^32^ ^]^ Site VI is engaged, among others, by sea anemone toxins ATX‐II and ApB, scorpion α‐toxins and δ‐conotoxins, but the exact binding motif within the channel is not yet known, although a participation of the linking loop between Site III–Site IV of DIV is hypothesized.^[^ ^26^, ^33^ ^]^ Local anesthetics and pyrethroids bind to two sites distinct from Site I–Site VI, and more potential binding pockets are still being discovered.^[^ ^34^ ^]^

### 2.2 VGSC Structure and Binding to TTX

**Figure 4:**
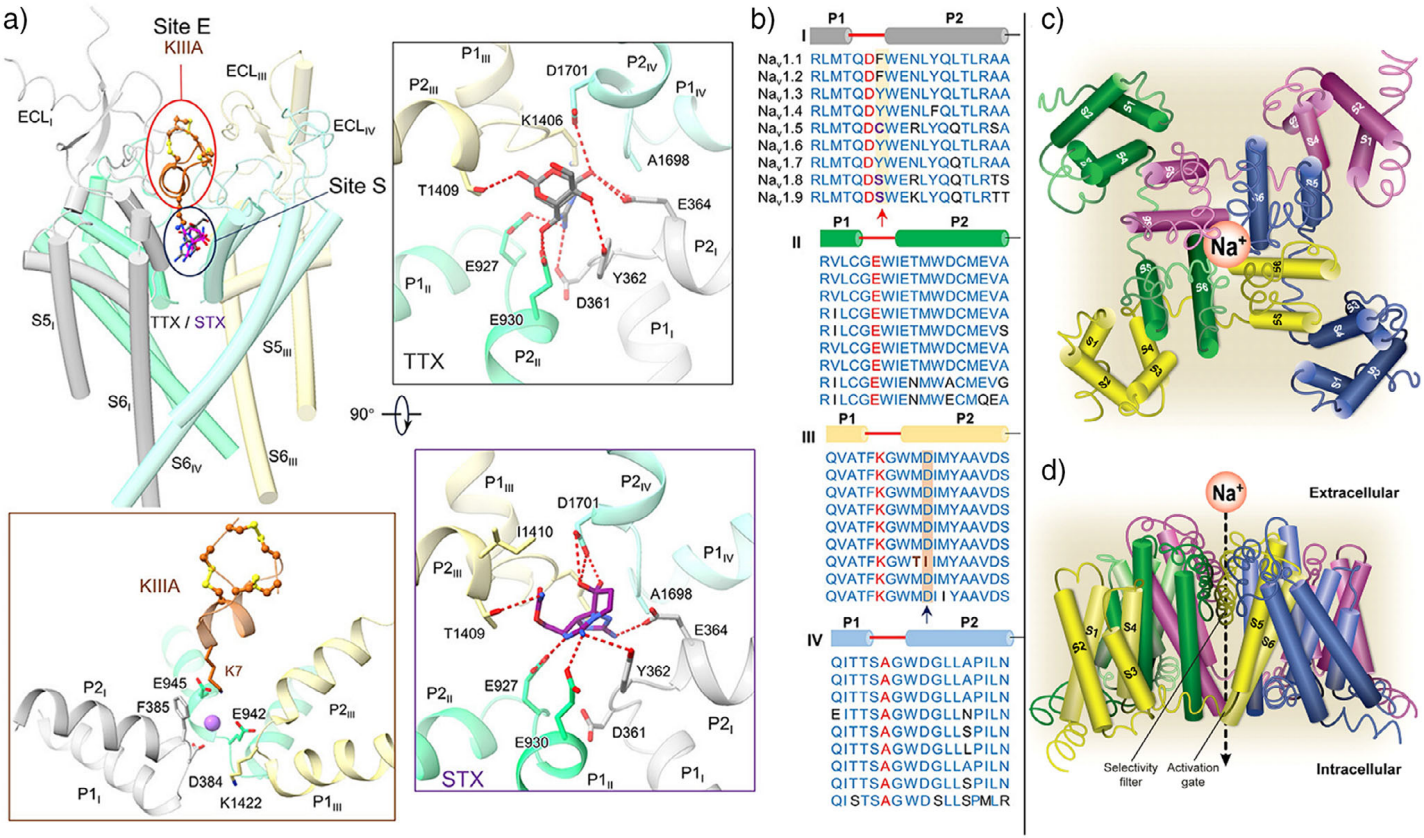
a) Simplified binding model of TTX (top right), STX (bottom right), KIIIA (bottom left), and superposition of all toxins (top left) with the interacting amino acids; b) Amino acid sequence compared between the different Na_V_ channels. Copyright a and b © 2023 Li, Wu, and Yan.^[^ ^39^ ^]^ Published by Informa UK Limited, trading as Taylor & Francis Group, used under Creative Commons BY‐NC 4.0.^[^ ^39^ ^]^ c) Top view on three‐dimensional representation with spatial orientation of the domain segments. The participation of each segment in the VSD and pore domain is highlighted. d) Side view on the α‐subunit with the distinct domains and segments assembling the ion channel and flow direction of the sodium ions. c, d) Each subunit within the tetramer is represented by a color. A related color scheme was used for each of the monomers in Figure 2. The segments that participate in the formation of the binding sites, Site I–Site VI, are denoted with S1–S6. Reprinted with permission.^[^ ^40^ ^]^

Several cryo‐EM structures unveil the binding mode of TTX (**1**) to VGSCs. The first available structural information was derived from Na_V_PaS, a VGSC from the cockroach *Periplaneta americana*.^[^ ^35^ ^]^ The cryo‐EM structure of the human Na_V_1.7 was recorded in the presence of TTX.^[^ ^35^, ^36^ ^]^ In general, ligand binding is facilitated by hydrogen bonds and salt bridges with acidic residues in the pore domains of segments I, II, and IV (Figure 4). These residues are unaltered in the TTX‐sensitive human VGSCs (hNa_V_1.1–1.4 and hNa_V_1.6–1.7).^[^ ^37^ ^]^ One major element that contributes to toxin binding and subsequent pore blockage is the selectivity filter at the extracellular entrance of the channel.^[^ ^35^, ^38^ ^]^ For example, the residues D361 (segment I) and E927 (segment II) of the DEKA motif bind to the guanidinium group, which additionally interacts with the acidic residues E364 (segment I), E930 (segment II), and a π−‐cation interaction with Y362 (segment I). Both the backbone amide and the side chain of T1409 (segment III) form hydrogen bonds with the C10 hydroxy group. The C8 alcohol is interacting with E930 (segment II), and the C4 alcohol is interacting with E364 (segment I). The C9 alcohol can form hydrogen bonds with both E927 (segment II) and the backbone of K1406 (DEKA motif).

Although binding of other toxins, such as HWTX‐IV or ProTx‐II, has been shown to impair conformational changes of the VSDs, there is no evidence that TTX binding has a major influence on the channel's conformation.^[^ ^41^ ^]^ TTX only alters local conformational rearrangements by a complex hydrogen bonding network, resulting in a blockage of the entrance to the selectivity filter.^[^ ^35^ ^]^ Although the discussed interactions are present in all available cryo‐EM structures, several other polar interactions can be found in individual structures, suggesting a highly complex interaction network. These findings are in accordance with the observation that the guanidinium moiety, the oxygen bridges, as well as the C4, C8, C9, C10, and C11 alcohols of TTX (**1**) are important for binding to the channel and that their removal results in a reduction of affinity by orders of magnitude.^[^ ^42^ ^]^ Of the nine VSGC, Na_V_1.5, Na_V_1.8, and Na_V_1.9 are inherently resistant to TTX. This feature arises from changes in the amino acid sequence at a single position in domain I. TTX‐sensitive channels contain a tyrosine above the selectivity filter that interacts via a π‐cation interaction with the guanidinium subunit, whereas resistant VGSCs host a cysteine (Na_V_1.5) or serine (Na_V_1.8 and Na_V_1.9) at the same position (Figure 4).

By selectively mutating C374 to tyrosine in the cardiac sodium channel Na_V_1.5, which establishes the composition of TTX‐sensitive VGSC, the sensitivity toward TTX (and STX) increased 730‐fold.^[^ ^43^ ^]^ To target both Na_V_1.8 and Na_V_1.9 in a similar manner as Na_V_1.7, the TTX core structure must be modified as exemplified by the generation of the Na_V_1.6‐selective probe 4,9‐anhydroTTX (**6**).^[^ ^44^ ^]^ This selectivity has been revealed by cryo‐EM combined with MD simulations. The binding site of Na_V_1.6 is nearly identical to Na_V_1.7, but small conformational changes were observed: 4,9‐anhydroTTX (**6**) binds 1.4 Å deeper in the Na_V_1.6 cavity than TTX (**1**) in Na_V_1.7. Additional conformational analyses of the sodium channel upon binding to 4,9‐anhydroTTX (**6**) revealed that the toxin has multiple possible conformations to bind to Na_V_1.7; only one dominates in Na_V_1.6. Deeper investigations showed that the amino acid residues E373, E377, and E939 contribute mainly to the binding of 4,9‐anhydroTTX (**6**). The helix containing those residues was found to be more flexible in Na_V_ 1.7, which impeded stabilizing the binding of 4,9‐anhydroTTX (**6**).^[^ ^44^ ^]^

In contrast to the mammalian VGSC, the bacterial Na_V_Ab is not affected by TTX (**1**) due to a modified selectivity filter.^[^ ^45^ ^]^ In this case, the DEKA motif is replaced by an EEEE ring within the pore.^[^ ^46^ ^]^ The higher negative charge density of the Na_V_Ab filter results in an enhanced affinity for sodium ions, which thereby outcompete TTX (**1**) for binding.^[^ ^38^ ^]^ Remarkably, the Na_V_Ab channel retains its selectivity for sodium ions, while replacing the DEKA motif by an EEEE ring in the human channel results in a Ca^2+^ selectivity. TTX‐bearing species, as well as their predators, show a high diversification of VGSC. For example, Na_v_1.4 of pufferfish hosts substitutions of aromatic for non‐aromatic amino acids, such as Asn or Cys instead of Tyr in the pore loop of domain I, which reduces TTX sensitivity by a factor of 2000.^[^ ^47^ ^]^ Other mutations have been identified in the pore region of all four domains, most of them in domain IV with homolog and paralog mutations for different members of VGSC and for different species, including newts, garter snakes, and mollusks.^[^ ^48^, ^49^, ^50^ ^]^ Although mutations within the pore region have the advantage of strongly reducing TTX sensitivity, they impair conductivity and sodium selectivity.^[^ ^51^ ^]^

TTX‐binding proteins have been identified in several TTX‐bearing species, suggesting a role in detoxification through the carriage and accumulation of TTX in certain tissues. The most prominent example is the *Pufferfish Saxitoxin and Tetrodotoxin Binding Protein* (PSTBP), which was found in different species of pufferfish.^[^ ^52^, ^53^, ^54^ ^]^ It is a carrier protein that transports TTX (**1**) among tissues such as the liver, the ovary, and the skin, thereby diminishing the concentration of free TTX (**1**) in the plasma.^[^ ^53^, ^55^, ^56^ ^]^

### 2.3 Role of VGSCs in Pain Signaling

Sensory neurons contain distinct sets of VGSCs, which are central to their excitability, but differ in their kinetics and activation threshold. Following the initial transduction of sensory stimuli, VGSCs initiate all‐or‐none action potentials via inward currents of sodium ions. These action potentials are propagated to the central nervous system, which finally lead to neurotransmitter release. The action potential is built by several VGSCs, starting with Na_v_1.3, Na_v_1.7, and Na_v_1.9, which amplify subthreshold stimuli. When reaching the threshold, the rising phase of the action potential is characterized by the activation of Na_v_1.6, Na_v_1.7, and Na_v_1.8, with the latter channel as the main contributor. Interestingly, Na_v_1.7 contributes to both the amplification of subthreshold stimuli and to the rising phase of the action potential. A more detailed description of VGSC's role in pain signaling is provided by Bennett et al.^[^ ^57^ ^]^

According to the varying response toward the chemical probe, VGSCs are divided into TTX‐sensitive (TTX‐s) and TTX‐resistant (TTX‐r) forms.^[^ ^2^ ^]^ TTX‐sensitive VSGC can be blocked by nanomolar concentrations of TTX, whereas resistant forms are blocked by micromolar concentrations. The latter include Na_V_1.5, Na_V_1.8, and Na_V_1.9. Seven of the nine VGSC subtypes (Na_V_1.1–Na_V_1.3, and Na_V_1.6–1.9) are expressed in dorsal root ganglia (DRG) neurons, which are located in the peripheral nervous system (PNS) and transmit pain signals from the skin and tendons to the central nervous system (CNS). These subtypes are thus potentially involved in conveying noxious stimuli and may represent a target for pain treatment. The main actors of the anatomical and physiological integrity of pain signaling are Na_V_1.7, Na_V_1.8, and Na_V_1.9, which have been genetically proven to be linked to human pain disorders.^[^ ^57^ ^]^ However, the high expression of Na_V_1.7 in DRG neurons, combined with multiple reported mutations that induce genetic‐hypoalgesic and hyperalgesic disorders, makes this subtype one of the most promising targets to alleviate pain.^[^ ^58^, ^59^ ^]^ Na_V_1.3 is typically expressed in early postnatal periods, and very low levels are detected in sensory neurons of adults.^[^ ^57^, ^60^ ^]^ Upon neural injuries such as nerve transection, ligation of the spinal nerve, or diabetic neuropathy, the expression is rapidly upregulated. As such, drugs with a high selectivity for the TTX‐s Na_V_1.3 are envisaged to allow highly selective treatment of these specific types of neuropathic pain.^[^ ^61^ ^]^

Na_V_1.8 and Na_V_1.9 are found in nociceptors and, together with Na_V_1.7, are expressed in DRG neurons, where the three subtypes mediate pain. Na_V_1.8 exhibits slow activation as well as inactivation and accounts for the majority of the inward current necessary to the rising phase of action potentials.^[^ ^62^ ^]^ Na_V_1.8 mRNAs were also detected and quantified in astrocytes, Müller glia, endothelial cells, fibroblasts, keratinocytes, and T lymphocytes. This subtype has been reported to contribute to inflammatory pain and neuropathic pain associated with acquired immunodeficiency syndrome (AIDS), diabetes, and cancer. SCN10A gain‐of‐function mutations are moreover associated with small fiber neuropathy and inherited erythromelalgia. Na_V_1.9 plays a major role in **1)** inflammatory, heat and mechanical pain hypersensitivity, as revealed in both (sub)acute and chronic inflammatory pain models; **2)** in the maintenance of bone cancer pain (together with the Na_V_1.8 subtype); **3)** the perception of cold pain under normal and pathological conditions; and **4)** visceral pain. More recently, multiple Na_V_1.9 genetic mutations were linked to painless or painful phenotypes. Specifically, congenital SCN11A loss‐of‐function mutations, such as congenital insensitivity to pain as well as type VII hereditary sensory and autonomic neuropathy, were reported to result in genetic diseases with a complete absence of pain.

### 2.4 VGSCs as Drug Targets

The findings discussed above make Na_V_1.8 the second most sought‐after target‐of‐interest (after the Na_V_1.7 subtype) to treat pain. Suzetrigine, a Na_V_1.8‐selective small molecule inhibitor developed by Vertex, has completed two phase 3 clinical trials and was approved as a first‐in‐class Na_V_‐targeted analgesic at the beginning of 2025.^[^ ^63^ ^]^ Selective blocking of neuronal action potentials is achieved by targeting TTX‐s VGSCs, specifically Na_V_1.1–1.4 and Na_V_1.6–1.7, which are predominantly present in skeletal muscles, neurons, and nerve fibers.^[^ ^57^ ^]^ This targeted blocking allows for the suppression of neuronal activity without interfering with cardiac signaling pathways. The significance of Na_V_1.7, a crucial channel involved in nociceptive signaling, has captured the attention of the pharmaceutical industry. Arylsulfonamides as potential inhibitors of Na_V_1.3 and Na_V_1.7, which are reported to target three different sites of the VSD IV,^[^ ^64^ ^]^ were investigated by, e.g., Pfizer, Genentech, as well as Xenon Pharmaceuticals and were entered into clinical trials, which were ultimately discontinued, presumably for a lack of target engagement. Wex Pharmaceuticals has conducted clinical trials to explore the use of TTX (**1**), marketed prior as Tectin, administered for the treatment of severe cancer‐related and chemotherapy‐related neuropathic pain. Their successful endeavors in phase 1 and phase 2 clinical trials^[^ ^65^ ^]^ have spurred further research aimed at optimizing formulations of the channel blocker and investigating structure–activity relationships (SAR) to enhance binding efficacy and selectivity for hNa1.7. A new formulation marketed under the name Halneuron was entered into phase 3 clinical trials.^[^ ^66^, ^67^ ^]^ These enhanced formulations may involve combinations with chemical permeation enhancers, vasoconstrictors, local anesthetics, or the utilization of targeted delivery systems. SiteOne Therapeutics is currently engaged in SAR studies on saxitoxin‐derived Site I‐selective sodium channel blockers.^[^ ^68^, ^69^, ^70^ ^]^

## 3 Distribution and Origin of Tetrodotoxin

**Figure 5:**
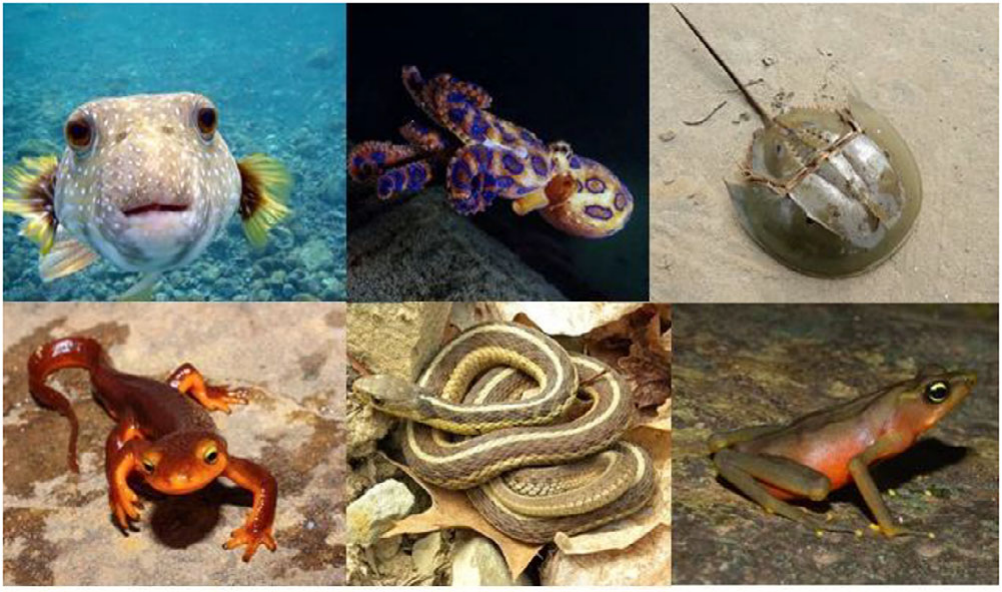
Examples of TTX‐bearing species. From top left to bottom right: *Arothron hispidus* (Pufferfish),^[^ ^80^ ^]^ *Hapalochlaena maculosa* (Blue‐Ringed Octopus),^[^ ^81^ ^]^ *Tachypleus gigas* (Horseshoe Crab),^[^ ^82^ ^]^ *Taricha torosa* (California Newt),^[^ ^83^ ^]^ *Thamnophis sirtalis* (Common Garter Snake),^[^ ^84^ ^]^ and *Atelopus limosus* (Limosa Harlequin Frog).^[^ ^85^ ^]^ All pictures reprinted with permission.

TTX (**1**) is found in a variety of phylogenetically unrelated terrestrial and aquatic species (Figure 5) such as pufferfish, blue‐ringed octopuses, horseshoe crabs, newts, frogs, gobies, xanthid crabs, gastropods, starfish, flatworms, ribbon worms, annelids, arrow worms, red calcareous algae, dinoflagellates, and bacteria.^[^ ^71^, ^72^ ^]^ For many species, TTX (**1**) serves as a defensive agent and is concentrated in the skin.^[^ ^73^, ^74^ ^]^ During the spawning season, the marine pufferfish increasingly transfer the neurotoxin to their eggs as protection for the offspring.^[^ ^75^ ^]^ This could be linked to the observation that TTX (**1**) also serves as a pheromone of female pufferfish (F*ugu niphobles*) to attract males.^[^ ^76^ ^]^ To avoid this defensive mechanism and to prey on the rough‐skinned newts, the common garter snake has evolved a resistance against intoxication.^[^ ^77^, ^78^ ^]^ The blue‐ringed octopus uses TTX (**1**) as a hunting tool to paralyze its prey.^[^ ^79^ ^]^ Therefore, it is located in their posterior salivary glands and transmitted through the saliva by biting the victim.

It is worth noting that TTX‐bearing species are distributed worldwide in diverse terrestrial, marine, fresh‐water, and brackish water environments (Figure 5). For instance, TTX‐containing newts were found in North America^[^ ^86^ ^]^ (*Notophthalmus viridescens and Taricha torosa*), Japan^[^ ^87^ ^]^ (*Cynops pyrrhogaster*), Germany^[^ ^86^ ^]^ (*Triturus spp*.), and Italy^[^ ^86^ ^]^ (*Triturus alpestris*). The geographical distribution of this potent neurotoxin in evolutionary diverse animals led to three hypotheses regarding its biochemical origin: exogenous TTX sources, endogenous TTX synthesis, and symbiotic relationships with TTX‐producing microorganisms. Many marine bacteria, such as *Pseudomonas spp*. and *Vibrio spp*., are reported to produce TTX (**1**), which could be accumulated through the food chain.^[^ ^88^, ^89^, ^90^, ^91^ ^]^ This hypothesis is based on the effect that pufferfish (*Takifugu rubripes*) were non‐toxic until the consumption of TTX‐containing organisms.^[^ ^92^ ^]^ In addition, the newt *Notophthalmus viridescens* is subject to considerable toxin level variations depending on their geographic origins, and it has been shown that these species lose their toxicity when fed with a TTX‐free diet.^[^ ^93^ ^]^ Examples that support the endogenous origin are the newt *Taricha granulosa* and the toad *Atelopus oxyrhynchus*, which increase their toxicity when kept in captivity without an external TTX (**1**) source.^[^ ^1^, ^73^, ^94^ ^]^ Bacterial symbiosis is a common phenomenon in marine animals, and, in many cases, bacterial secondary metabolites have been found in the host animal species.^[^ ^95^ ^]^ The symbiosis‐based hypothesis is supported by the observation that TTX‐producing *Vibrio* strains could be cultured from the intestine of a Xanthid crab (*Atergatis floridus*).^[^ ^90^ ^]^ In addition, gut microbiota structures were significantly different between toxic and non‐toxic pufferfish.^[^ ^96^ ^]^ A symbiotic bacterial strain, which was isolated from Japanese pufferfish (*Fugu rubripes*) named He‐1 was found to produce TTX (**1**). The subculture strain He‐2 doesn´t produce TTX (**1**) under normal cell culture conditions, only upon adding a small peptide metabolite from He‐1, which underlines the interconnection of bacterial symbionts related to TTX (**1**) biosynthesis.^[^ ^97^ ^]^

## 4 Tetrodotoxin Natural Product Family

**Scheme 1:**
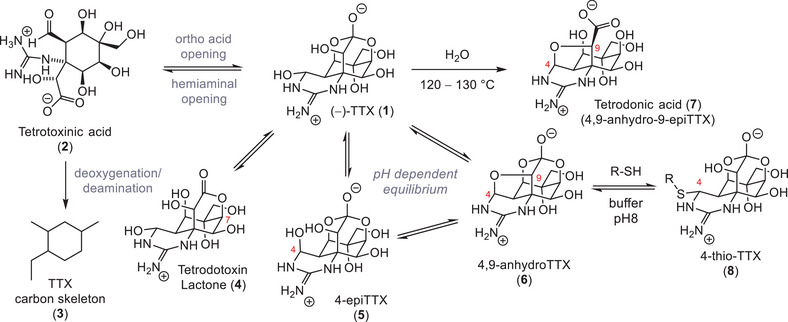
Chemical equilibrium of TTX (**1**) and the underlying carbon skeleton (**3**).

Tetrodotoxin (**1**, Scheme 1) is the most prominent member of a large natural product family.^[^ ^72^ ^]^ Its structure (**1**) is characterized by a highly functionalized dioxaadamantane core appended to a six‐membered guanidine heterocycle, which is formed via a hemiaminal. Disconnecting the spontaneously forming C4 *N*,*O*‐acetal and C10 ortho acid leads to tetrotoxinic acid (**2**), which is known to exist in solution.^[^ ^98^ ^]^ This aminopolyol (**2**) contains seven stereogenic centers, including an α‐tertiary amine, an α‐hydroxy acid, and a range of 1,2‐diol moieties. Stripping the molecule (**2**) down to the basic carbon skeleton (**3**) emphasizes the simplicity of the backbone, which stands in sharp contrast to the high density of functional groups on TTX (**1**). Many members of the TTX natural product family are TTX isomers and/or in equilibrium with the neurotoxin. Opening of the C10 ortho acid functionality, for instance, produces almost exclusively the C7 lactone form of tetrodotoxin (**4**).^[^ ^7^ ^]^ In acidic solutions, the epimerization of the C4 hemiaminal provides 4‐epiTTX (**5**), whereas condensation of the C9 alcohol with the hemiaminal produces 4,9‐anhydroTTX (**6**).^[^ ^99^ ^]^ Heating TTX (**1**) to 120–130 °C in water epimerizes the C9 stereocenter, which facilitates opening of the C10 ortho acid and the formation of an ether bridge between C4 and C9 to give tetrodonic acid (4,9‐anhydro‐9‐epiTTX, **7**).^[^ ^6^, ^7^, ^98^ ^]^ The analogues 4‐epiTTX (**5**), 4,9‐anhydroTTX (**6**), and tetrodonic acid (**7**) are often isolated together with TTX (**1**) from natural sources^[^ ^100^ ^]^ such as the newt species *Cynops ensicauda* ^[^ ^93^, ^101^ ^]^ or the frog species *Brachycephalus ephippium*.^[^ ^74^, ^102^ ^]^ It was shown that the equilibrium between TTX (**1**), 4‐epi‐TTX (**5**), and 4,9‐anhydroTTX (**6**) is pH dependent and that subjecting 4,9‐anhydroTTX (**6**) to thiols at pH 8 leads to the formation of 4‐thioTTX (**8**) derivatives.^[^ ^99^ ^]^

**Figure 6:**
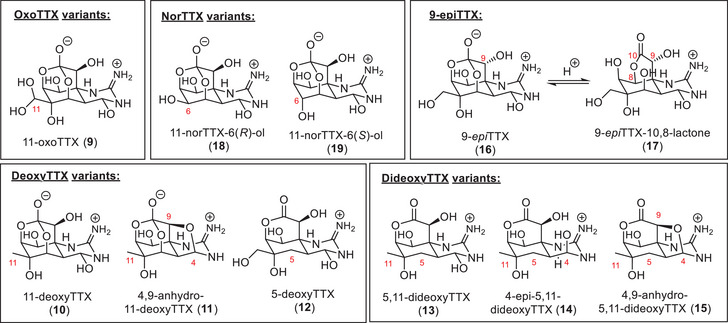
Selection of TTX derivatives isolated from natural sources.

In addition to TTX isomers (Scheme 1), which include epimers with rearranged core structures (epiTTX), a range of oxidized (oxoTTX), deoxygenated (deoxyTTX), and demethylated (norTTX) analogues are commonly isolated from natural sources (Figure 6). The oxidized 11‐oxoTTX (**9**) was found in pufferfish (*Arothron nigropunctatus*),^[^ ^103^ ^]^ newt (*Taricha spp*.),^[^ ^72^, ^104^ ^]^ frog (*Brachycephalus ephippium*),^[^ ^102^ ^]^ gastropod (*Nassarius* spp.),^[^ ^93^, ^105^ ^]^ and xanthid crab (*Atergatis floridus*)^[^ ^106^ ^]^ species. Monodeoxygenated 11‐deoxyTTX (**10**) and its interconvertible isomer 4,9‐anhydro‐11‐deoxyTTX (**11**) were isolated from the newt *Cynops ensicauda popei*.^[^ ^107^ ^]^ The 11‐deoxyTTX was also found in the frog (*Brachycephalus ephippium*)^[^ ^74^ ^]^ and pufferfish (*Fugu niphobles*)^[^ ^108^ ^]^ sources, where it was detected together with 5‐deoxyTTX (**12**). Dideoxygenated 5,11‐dideoxyTTX (**13**) stems from the pufferfish *Takifugu poecilonotus* ^[^ ^109^ ^]^ as well as the flatworm *planocerid sp*.^[^ ^109^, ^110^ ^]^ and is in equilibrium with 4‐epi‐5,11‐dideoxyTTX (**14**) and 4,9‐anhydro‐5,11‐dideoxyTTX (**15**), which was determined as part of their chemical synthesis.^[^ ^109^ ^]^ 9‐epiTTX (**16**) was isolated from *Takifugu flavipterus*.^[^ ^111^ ^]^ NMR studies suggested that it exists in an equilibrium as 9‐*epi*TTX hemilactal (**16**) and 9‐*epi*TTX‐10,8‐lactone (**17**).^[^ ^111^ ^]^ Both diastereomers of 11‐norTTX (**18** and **19**) were isolated from the sea slug *Pleurobranchaea maculata* ^[^ ^112^ ^]^ and the pufferfish *Lagocephalus sceleratus*.^[^ ^113^, ^114^ ^]^ Only the 11‐norTTX‐6(*R*)‐ol isomer (**18**) was isolated from the crab *Atergatis floridus* ^[^ ^106^ ^]^ and newt *Cynops ensicauda*,^[^ ^101^ ^]^ whereas solely the 11‐norTTX‐6(*S*)‐ol isomer (**19**) was isolated from the frog *Brachycephalus ephippium* ^[^ ^74^ ^]^ and the pufferfish *Arothron nigropunctatus*.^[^ ^115^ ^]^

**Figure 7:**
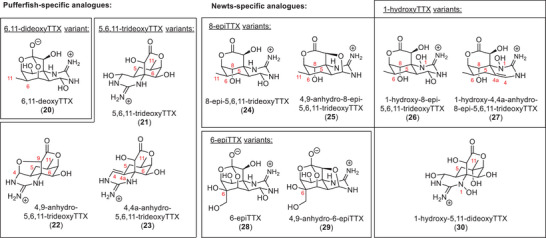
Comparison of TTX derivatives in pufferfish and newt species.

It is hypothesized that the biosynthesis of TTX and its analogues is dependent on the host species.^[^ ^1^, ^66^ ^]^ This theory is supported by the fact that many members of the family are only isolated from specific hosts. Consequently, a wealth of TTX‐derived structures was found with a high level of diversity regarding the substitution pattern on the natural product core structure. The biosynthetic differences could be highlighted by comparing the isolated TTX derivatives from pufferfish and newt species (Figure 7). The 6,11‐dideoxy and 5,6,11‐trideoxyTTX species that incorporate the (*R*)‐configuration at C8, for example, were not found in newts but observed in pufferfish. They include 6,11‐dideoxyTTX (**20**) from *Fugu niphobles* ^[^ ^108^ ^]^ 5,6,11‐trideoxyTTX (**21**), 4,9‐anhydro‐5,6,11‐trideoxyTTX (**22**), and 4,4a‐anhydro‐5,6,11‐trideoxyTTX (**23**) from *Fugu poecilonotus*.^[^ ^116^, ^117^ ^]^ 8‐EpiTTX, 1‐hydroxyTTX, and 6‐epiTTX variants were only isolated from newts. *Cynops ensicauda popei* ^[^ ^107^ ^]^ was the source of 8‐epi‐5,6,11‐trideoxyTTX (**24**), 4,9‐anhydro‐8‐epi‐5,6,11‐trideoxyTTX (**25**), 1‐hydroxy‐8‐epi‐5,6,11‐trideoxyTTX (**26**), 1‐hydroxy‐4,4a‐anhydro‐5,6,11‐trideoxy‐TTX (**27**), and 6‐epiTTX (**28**). *Cynops pyrrhogaster* ^[^ ^87^ ^]^ was the source of 4,9‐anhydro‐6‐epiTTX (**29**) and *Taricha granulosa* ^[^ ^118^ ^]^ was the source of 1‐hydroxy‐5,11‐dideoxyTTX (**30**).

**Figure 8:**
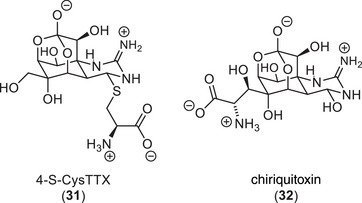
Naturally occurring amino acid‐containing TTX derivatives.

Two members of the natural product family have amino acid structures incorporated: 4‐S‐CysTTX (**31**) from the puffer fish *Fugu pardalis* ^[^ ^119^, ^120^ ^]^ and chiriquitoxin (**32**) from the toad *Atelopus chiriquiensis* ^[^ ^120^, ^121^, ^122^ ^]^ (Figure 8). 4‐S‐CysTTX was isolated from the puffer fish liver, and it was experimentally validated that it could be prepared through a reaction of 4,9‐anhydroTTX (**6**) with cysteine in aqueous solutions (pH 8). This has led to the hypothesis that it is a metabolic product, which is formed non‐enzymatically.

**Figure 9:**
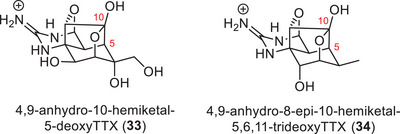
Hemiketal derivatives of TTX isolated from newts.

The latest additions to the TTX family were the C5, C10 carbon‐carbon bonded 4,9‐anhydro‐10‐hemiketal‐5‐deoxyTTX (**33**) and 4,9‐anhydro‐8‐epi‐10‐hemiketal‐5,6,11‐trideoxyTTX (**34**) that were isolated from the newt *Cynops ensicauda popei* (Figure 9).^[^ ^123^ ^]^ Their isolation has led to the hypothesis that the biosynthesis of TTX (**1**) in newts proceeds through C5, C10 carbon–carbon bonded species and that 4,9‐anhydro‐10‐hemiketal‐5‐deoxyTTX (**33**) is the direct precursor to 4,9‐anhydroTTX (**6**). The isolation of new members of the TTX natural product family strongly contributes to the elucidation of the species‐specific biosynthesis of TTX (**Chapter 5**) and is therefore still the focus of modern research.^[^ ^66^ ^]^

## 5 Biosynthesis of TTX

**Scheme 2:**
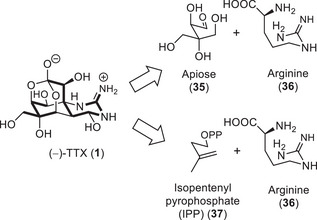
Early hypotheses of the TTX (**1**) biosynthesis. Bold segments correspond to the carbons sourced from each of the two building blocks.

To date, many hypotheses for the biosynthesis of TTX (**1**) have been discussed based on structural features of the TTX natural product family members.^[^ ^124^ ^]^ An early proposal suggested that TTX could be traced back to a branched pentose such as apiose (**35**) and the amino acid arginine (**36**) (Scheme 2).^[^ ^118^ ^]^ This hypothesis accounts for the full oxygenation pattern as well as the guanidine moiety. However, the isolation of many deoxyTTX derivatives suggests that the TTX core is built from less oxygenated precursors such as isopentenyl pyrophosphate (**37**) followed by late‐stage oxidations.^[^ ^125^ ^]^

**Scheme 3:**
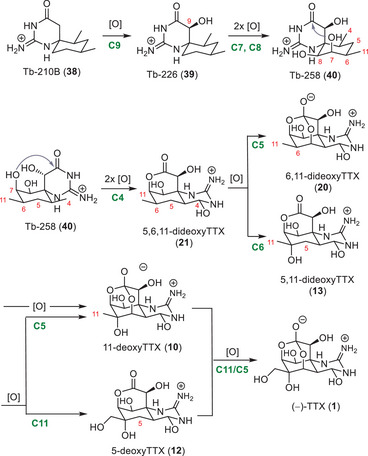
Proposed biosynthesis of TTX (**1**) in marine species by sequential oxidation.

It is reasonable to assume that the biosynthesis of TTX (**1**) in marine and terrestrial taxa follows distinct pathways,^[^ ^1^, ^66^ ^]^ based on the structural differences between the members of the TTX natural product family that stem from pufferfish and newt sources (see **Chapter 4**). The isolation of mixtures of 5‐deoxyTTX (**12**), 11‐deoxyTTX (**10**), 5,11‐dideoxyTTX (**13**), 6,11‐dideoxyTTX (**20**), and 5,6,11‐trideoxyTTX (**21**) in the pufferfish hints at a stepwise biosynthetic late‐stage oxidation from 5,6,11‐trodeoxyTTX (**21**) to TTX (**1**) (Scheme 3).^[^ ^109^ ^]^ This hypothesis is supported by the identification of enzymes in the biosynthetic gene cluster of other paralytic shellfish toxins (PST), such as saxitoxin, that perform selective C–H oxidation reactions to install defined hydroxy groups.^[^ ^126^ ^]^ In addition, members of the natural product family with higher levels of oxidation than TTX (**1**) were discovered, such as 11‐oxoTTX (**9**).^[^ ^102^, ^109^ ^]^

A new perspective on the early stages of the TTX (**1**) biosynthesis in marine species arose through the isolation of the spirocyclic guanidinyl lactams Tb‐210B (**38**), Tb226 (**39**), and Tb‐258 (**40**) from the pufferfish *Tetraodon biocellatus* (Scheme 3).^[^ ^127^ ^]^ The guanidylated carbon skeleton (**38**) is thereby sequentially oxidized to 5,6,11‐trideoxyTTX (**21**). Isolated intermediates include C9 hydroxylated cyclohexane Tb‐226 (**39**), which could be further oxidized to Tb‐258 (**40**) via oxidation on C7 and C8. Oxidation of the C4 position, followed by lactam hydrolysis and esterification, leads to 5,6,11‐trideoxyTTX (**21**).

**Scheme 4:**
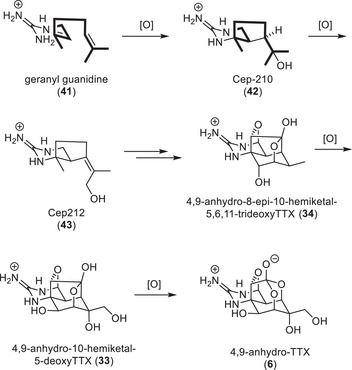
Proposed terpene‐based TTX (**1**) biosynthesis pathway in newts.

The structural features of TTX derivatives that stem from newt sources suggest a biosynthesis through a terpene pathway (Scheme 4). Determining the biosynthetic building blocks of TTX in newts was attempted by feeding [2–^14^C]‐acetate, [^14^C (U)]‐glucose, [guanido‐^14^C]‐arginine and [ureido‐^14^C]‐citrulline to *Taricha torosa* and *Taricha granulosa*, but did not lead to incorporation of the radioactive building blocks.^[^ ^128^ ^]^

The hypothesis of a different biosynthetic origin of TTX compared to maritime animals was supported by the isolation of cyclic guanidine‐containing monoterpenes (**42** and **43**) from the toxic Japanese sword‐tail newt (*Cynops ensicauda popei*) (Scheme 4).^[^ ^124^ ^]^ Oxidative cyclization of geranyl guanidine (**41**) may afford **42,** which could be further oxidized to **43**. These findings are further corroborated by the identification of the C5, C10 carbon–carbon bonded 4,9‐anhydro‐8‐epi‐10‐hemiketal‐5,6,11‐trideoxyTTX (**34**) and 4,9‐anhydro‐10‐hemiketal‐5‐deoxyTTX (**33**).^[^ ^123^ ^]^ These more elaborate TTX derivatives were also isolated from *Cynops ensicauda popei* and are therefore likely products from biosynthetic oxidations of Cep‐212 (**43**). In theory, 4,9‐anhydro‐10‐hemiketal‐5‐deoxyTTX (**33**) could be converted to 4,9‐anhydroTTX (**6**) by a Baeyer–Villiger‐type monooxygenase.^[^ ^123^ ^]^

## 6 Biological Activity of Tetrodotoxin Derivatives

### 6.1 General Considerations

The first systematic studies to understand the pharmacological action of TTX date back to the early 20th century. For example, Itakura studied the pharmacological action of TTX with living frogs and rabbits, whereas Ozaki utilized isolated sciatic nerves from frogs.^[^ ^129^, ^130^ ^]^ A growing understanding of electrophysiological principles over the ensuing decades and the invention of patch clamp technology marked major hallmarks in uncovering the exact molecular mode of action of TTX.^[^ ^131^ ^]^ Consequently, it was discovered that TTX exhibits high affinities for the so‐called TTX‐sensitive channels, Na_V_1.1–Na_V_1.4 and Na_V_1.6‐Na_V_1.7, and only shows micromolar activities on the TTX‐resistant channels, Na_V_1.5, Na_V_1.8, and Na_V_1.9.^[^ ^132^ ^]^ This high selectivity, together with the high potency, made it an invaluable instrument for the investigation of sodium currents activating the nerve action potential, which, among other things, helped elucidate the action potential mechanism and membrane currents by Hodgkin and Huxley.^[^ ^133^, ^134^, ^135^ ^]^ To this day, TTX remains the most widely used tool in neuroscience and electrophysiology for the suppression of action potentials. As such, safe handling is essential.

**Figure 10:**
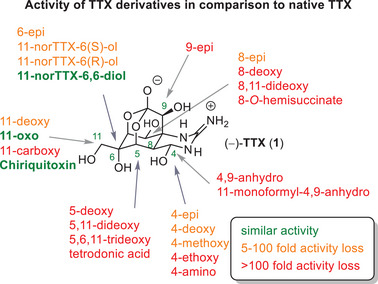
Relative potencies of TTX derivatives from different assays in comparison to internal TTX (**1**) standard; a detailed overview is compiled in the Supporting Information.

In the Supporting Information, a compilation of TTX activity data reported in the literature was assembled. Comparing data from different literature sources presents a challenge due to varying assay principles and conditions.^[^ ^136^ ^]^ In addition, differences in the amino acid sequences of individual Na_V_ subtypes across species, e.g., frog or rat versus human, add yet another layer of complexity. However, the reviewed biological activity data on the SAR of TTX derivatives included unmodified TTX (**1**) as a positive control, allowing for direct comparison. Thus, the activity data were summarized as relative potencies compared to TTX (Figure 10), illustrating the resulting activity loss or gain where possible. It is worth noting that, for assays carried out in primary tissues or cultures thereof, multiple TTX‐sensitive Na_V_ family members can aid the observed potency data. Nevertheless, the provided analysis can contribute to the overall understanding of the SAR of TTX (**1**) and its derivatives, even in the absence of a clear differentiation for drug‐relevant targets such as Na_V_1.7.

Throughout the past decades, a wide array of research groups has extensively reported on the potencies of TTX derivatives, encompassing changes in most conceivable positions of the TTX backbone. The activity data were obtained through various sources, including Na_V_ competition binding assays with radiolabeled TTX or STX in cell lines overexpressing specific family members, as well as from intact tissue preparations. Additionally, cell toxicity assays were utilized.^[^ ^137^ ^]^ However, in most cases, the expression profile of individual Na_V_s in the particular tissue investigated is not reported. Over the past years, a clear shift has been observed toward the utilization of electrophysiological assays detailing the potencies for distinct members of the Na_V_ channel family.^[^ ^138^ ^]^ This advancement offers invaluable insights into the safety profile of TTX derivatives and, ultimately, ascertains whether TTX can serve as a promising starting point in a drug discovery campaign.

### 6.2 SAR Profiles of TTX Derivatives

The pharmacophore of TTX consists of a highly oxidized, adamantane‐shaped scaffold flanked by a prominent guanidinium group. It has been hypothesized in several studies that the guanidinium anchor locks the TTX scaffold in the Na_V_ pore at Site I and is stabilized by several hydrogen bonding interactions.^[^ ^28^ ^]^ Given the low molecular weight of this small molecule toxin (MW = 319 g mol^−1^) and the single‐digit nanomolar potency on specific Na_V_s across several assays, its ligand efficiency (LE) is very high. LE, a metric used in drug discovery to identify lead compounds with optimal combinations of physicochemical and pharmacological properties, evaluates the binding efficiency of a ligand to its target. It is defined as the ratio of the binding energy (Gibbs free energy, ΔG) to the number of non‐hydrogen atoms in the ligand.^[^ ^139^, ^140^, ^141^ ^]^ Therefore, TTX is highly effective in binding to its target relative to its size.

Analyzing the available assay data and relative potencies of TTX derivatives reveals that the SAR is quite steep for several positions. Even slight modifications, such as epimers of hydroxy groups, result in a loss of activity by more than 1000‐fold compared to the parent compound TTX (Figure 10). The analyzed examples indicate that many TTX atoms already occupy the optimal position in the binding pocket. As a result, even small modifications and deviations can significantly impact the binding strength. Although the 4‐position shows some examples with moderate loss, the positions C5, C8, and C9 appear to be critical for maintaining potency.^[^ ^142^ ^]^ However, the positions C6 and C11 seem to tolerate a broad range of modifications, resulting in only partial loss of activity, as seen with 6‐epiTTX (**28,** Figure 7), potency retention for chiriquitoxin^[^ ^143^, ^144^ ^]^ (**32**, Figure 8), and even up to 5‐fold potency increase for 11‐oxoTTX (**9,** Figure 6), which was confirmed by two independent assays.^[^ ^144^, ^145^ ^]^

Available cryo‐EM structures indicate that the 11‐position points toward the solvent‐exposed exit vector, offering a useful handle for derivatization and functionalization. Mari Yotsu‐Yamashita's group studied the binding mode of the C11 hydroxyl group, supported by thermodynamic mutant cycle analysis in rat Na_V_1.4, and reported on the key interactions in this pore region.^[^ ^146^ ^]^ For example, 11‐carboxyTTX (**44**) is too polar and not tolerated, while chiriquitoxin (**32**), a glycine‐modified TTX analog, shows that shifting the polar group further along the exit vector on top of the TTX scaffold contributes positively to binding.^[^ ^121^ ^]^

The unique case of 4,9‐anhydroTTX (**6**) illustrates how minimal structural modifications, such as the nominal loss of a single water molecule, can significantly alter both the potency and selectivity across various Na_V_ channels. For example, this derivative exhibits a significant reduction in potency compared to TTX (**1**) on Na_V_1.7 (an 80‐fold decrease, from 17 to 1340 nM) and Na_V_1.2 (a 53‐fold decrease, from 5 to 258 nM). However, it remains potent on Na_V_1.6 (a 26‐fold decrease, from 2 to 52 nM),^[^ ^44^ ^]^ making it an intriguing probe molecule for this Na_V_ channel (see Supporting Information for details). An earlier study^[^ ^42^ ^]^ reported an even more pronounced shift in favor of Na_V_1.6 selectivity. Researchers have long been intrigued by this divergence, and only recently, the binding mode of 4,9‐anhydroTTX (**6**) has been described by the groups of Zhao, Huang, and Jiang.^[^ ^44^ ^]^ The authors discovered a dynamic binding effect that goes beyond the pure sequence identity of the pocket, which is highly conserved between the individual Na_V_ channels with only minor alterations. As such, accurately predicting which molecules will selectively bind to specific Na_V_ channels remains a challenge.

### 6.3 Notable TTX Derivatives

In recent years, many new TTX derivatives, including potential biosynthetic precursors, such as Cep‐210 (**42**) and Cep‐212 (**43**), have been discovered (Scheme 4).^[^ ^124^, ^147^ ^]^ Often, the isolated amounts of these natural products only allow for structure elucidation and not further testing in biological assays. However, a better understanding of how these small molecules bind to the Na_V_ channels and their selectivity profiles across the different subtypes is highly desired and will contribute to future drug discovery programs using TTX (**1**) as a lead molecule.

**Figure 11:**
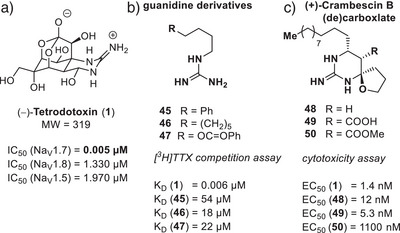
Reported SAR studies potentially addressing the TTX (**1**) binding site. a) TTX (**1**) potency extracted from Rosker et al.^[^ ^42^ ^]^; b) guanidine derivatives^[^ ^148^ ^]^; c) (+)‐crambescin B (de)carboxylate derivatives: the expected structural similarity to TTX (**1**) does not result in a comparable binding mode.^[^ ^149^ ^]^

A pioneering study from 1979 postulated that the guanidinium component is the essential element of TTX (**1**) binding and conducted tests on several mono‐linked guanidinium derivatives (**45**–**47**, Figure 11).^[^ ^148^ ^]^ Utilizing a [^3^H]TTX‐based competition binding assay, they identified a set of different TTX derivatives with potencies in the lower micromolar range (18–54 µM). Although the authors conducted meticulous control experiments, they could not unambiguously rule out the possibility that non‐specific surface charge perturbations near the channel, potentially induced by positively charged guanidiniums (chaotropic effect), may occur.

Another notable example involves the crambescin natural product family, as reported in 2016 by the Nishikawa group.^[^ ^149^ ^]^ The central scaffold of the investigated crambescin analogs (**48**–**50**) is formed by a 6‐membered cyclic guanidinium‐containing motif, and the authors underscored its potential three‐dimensional similarity to TTX (**1**). Activities of these crambrescin analogs were assessed by a cytotoxicity assay (Figure 11). This assay is based on the principle that the cytotoxic effects of veratridine, a channel opener, in the presence of ouabain, an Na^+^/K^+^ ATPase inhibitor, can be antagonized by TTX (**1**).^[^ ^137^ ^]^

The observed effects were not evoked based on the blockage of Site I within the VGSC but appeared to be driven by the Na_V_ veratridine binding site (Site II) or potentially other mechanisms.^[^ ^150^ ^]^ The provided examples clearly illustrate the challenge of identifying TTX (**1**)‐inspired small molecules or guanidine derivatives that exhibit comparable high potency in blocking specific Na_V_ channels, although high‐throughput screening methodologies for ion channels and nociceptors exist.^[^ ^151^, ^152^ ^]^

### 6.4 Tetrodotoxin‐Based Chemical Probes and Biochemical Tool Compounds

Since the seminal reports in 1979, there has been a marked interest in the development of chemical probes based on the TTX (**1**) scaffold to further elucidate the biology of Na_V_ channels. For example, the exploration of fluorescently (**51**–**53**) and radio‐labeled (**51**, **54**) probes,^[^ ^145^, ^153^ ^]^ as well as the conjugation of photoactivatable^[^ ^154^, ^155^, ^156^, ^157^ ^]^ groups (**51**) to TTX (**1**) resulting in irreversible binding to Na_V_ channels, have facilitated the study of prolonged suppression of action potentials. These advancements hold significant potential for the future of this field for specific competition binding assays at Site I, Na_V_ channel imaging or mapping, and safe toxin handling for purification.^[^ ^158^ ^]^

**Figure 12:**
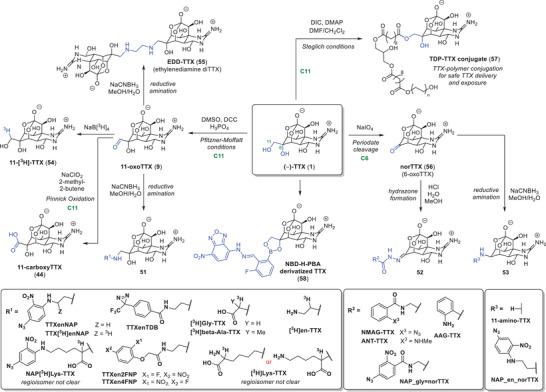
Modification of TTX (**1**)’s positions C6 and C11 yields a variety of chemical probes and tool compounds. Reagents: DIC = *N,N“*‐diisopropylcarbodiimide, DCC = *N,N”*‐dicyclohexylcarbodiimide, DMAP = 4‐dimethylaminopyridine. TTX (**1**) derivatives: AAG = 2‐azidoanthraniloylglycine hydrazide, Ala = alanine, ANT = anthraniloyl hydrazide, EDD‐TTX = ethylenediamine diTTX, en = ethylenediamine, 4FNP = 4‐fluoronitrophenol, 2FNP = 2‐fluoronitrophenol, Gly = glycine, Lys = lysine, NAP = nitro‐azidophenyl, NBD‐H‐PBA TTX = 4‐hydrazino‐7‐nitro‐2,1,3‐benzoxadiazole phenyl boronate TTX, NMAG = *N*‐methylanthraniloylglycine hydrazide, TDB = trifluoromethyl diazirinobenzoyl, TDP = biocompatible and biodegradable polymer poly(triol dicarboxylic acid)‐co‐poly(ethylene glycol).

Based on the SAR of TTX (**1**) derivatives, the most promising modifications and elongations predominantly take place at the solvent‐exposed vector, specifically at positions C11 and C6 (Figure 12, see Supporting Information for activity data). This has led to intriguing compounds, such as a TTX dimer, named EDD‐TTX (**55**), connected by an ethylenediamine linker at C11. The authors report that the binding properties (K_D_) were found to be similar to those of native TTX (**1**).^[^ ^159^, ^160^ ^]^ This finding is remarkable as the molecule's size and complexity increase.

In terms of synthetic accessibility, the Pfitzner–Moffatt oxidation^[^ ^161^ ^]^ is the most commonly applied technique to oxidize the C11 hydroxyl group of TTX (**1**), yielding 11‐oxoTTX (**9**) as a central intermediate and achieving yields of up to 35%.^[^ ^153^ ^]^ High yields are crucial to maximize output and minimize the amount of toxic TTX (**1**) used in the synthesis, as reported by several groups.^[^ ^157^ ^]^ Subsequent reductive amination provides a straightforward method to attach a diverse set of reporter molecules to generate tailored chemical probes.

Conversely, periodate cleavage has been used to access the C6 ketone‐functionalized 6‐oxoTTX (**56**), reducing the linker length by one carbon unit. It is noteworthy that a broad range of groups were tolerated at the studied positions (dye derivatives and photoactivatable variants), suggesting ample room for probe attachments.^[^ ^154^, ^162^ ^]^ In addition to TTX‐derived probes, the Kohane group has reported a polymeric ester prodrug of TTX (**57**), specifically designed for its slow release.^[^ ^163^, ^164^ ^]^ Given that free TTX (**1**) has a relatively short duration of action (lasting only hours), a modification was implemented by attaching a biodegradable polymer to TTX at the C11 position through a Steglich esterification to give **57**. This polymer prodrug (**57**) facilitates the safe delivery and gradual release of TTX (**1**), even at doses that would significantly surpass the lethal dose if TTX (**1**) itself were administered by injection. This methodology could present an advantage over standard subcutaneous TTX (**1**) administration, as the polymer–tetrodotoxin conjugates have the capacity to induce prolonged local anesthesia with minimal toxicity. The family of polymers developed using this approach, including PEG and polyglycerol sebacate, holds the potential to produce in vivo nerve blocks with a duration of the activity ranking from hours to days, with minimal local and systemic toxicities. Lastly, Kawasue and Oshiro reported an innovative NBD‐H‐PDA dye‐modified TTX derivative (**58**) with an absorption maximum at 503 nm for sensitive analytical detection in natural extracts via HPLC.^[^ ^158^ ^]^

As a side note, the Du Bois group^[^ ^165^, ^166^ ^]^ has developed advanced dye‐modified probes based on STX with varying linker lengths and lipophilicities. STX binds to the same site as TTX (**1**), and derivatives thereof provide invaluable insights into the tolerated chemical space. The outcome for the STX probes revealed that the reporter groups greatly affected Na_V_ activity, resulting in a 10‐ to 30‐fold reduction in potency. However, the resulting probe molecules still maintain a potency of approximately 40–100 nM, which is adequate for use as probes. In contrast to STX, TTX (**1**) has a unique probe profile due to its different Na_V_ affinities. TTX (**1**) only exhibits micromolar affinity for the cardiac channel Na_V_1.5, unlike STX, which is active in the nanomolar range (see Supporting Information for details).

### 6.5 Other TTX Derivatives Functionalized at C4 Position

TTX (**1**) typically exists as an interconverting mixture of TTX (**1**), 4‐epiTTX (**5**), and 4,9‐anhydroTTX^[^ ^99^ ^]^ (**6**) in an acidic aqueous solution due to the chemical equilibrium between these compounds (Scheme 1). TTX's instability in water results in different derivatives, the composition of which depends on whether it is isolated from natural samples or synthesized. This compositional variability can impact assay results, making it essential to understand these mixtures for precise IC_50_ value determination. Previous studies have reported that the hemiaminal ether carbon within 4,9‐anhydroTTX (**6**) can react with an excess of thiol compounds, including cysteine, reduced glutathione (GSH), and mercaptoethanol. As a result, the C4 position of TTX (**1**) emerges as an appealing site for native functionalization, owing to its inherent reactivity. In fact, a cysteine substitution of TTX (**1**), known as 4‐S‐CysTTX (**31**) (Figure 8), has been isolated from the liver of the pufferfish *Takifugu pardalis*, suggesting it as a likely metabolite of TTX.^[^ ^120^ ^]^ Attempts have also been made to detoxify TTX (**1**) with thiols. However, the detoxification of TTX (**1**) with thiols may not prove successful due to instability, even though 4‐S‐CysTTX (**31**) exhibits almost no toxicity to mice. This chemistry has been used to synthesize TTX (**1**) conjugates with biotin, antibodies, and antigens (such as KLH) at position 4 via thiol‐maleimide click chemistry.^[^ ^167^ ^]^

## 7 Synthetic Approaches toward Tetrodotoxin

### 7.1 Total Syntheses of Tetrodotoxin

**Scheme 5:**
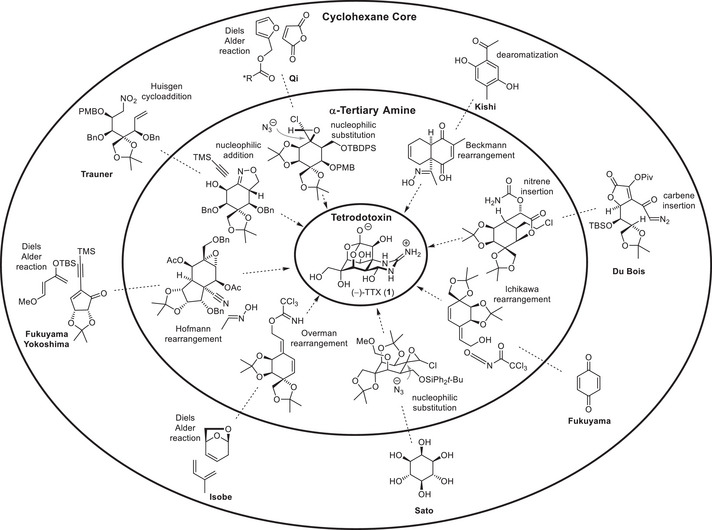
Synthetic approaches to TTX (**1**): Assembly of the cyclohexane core and α‐tertiary amine.

Until 2025, eight total syntheses of TTX (**1**) have been published. Over the past six decades since its structural elucidation, many synthetic methodologies have been developed that aimed at accessing unique features of this challenging target. The feasibility of these approaches was demonstrated in the total synthesis of TTX (**1**) and, in an effort to establish formal syntheses, key intermediates of prior routes were prepared by various groups. In this review, we are focusing on discussing all published total syntheses up to January 2025 as well as selected strategies that yielded natural and unnatural derivatives of TTX (**1**). The most striking formal syntheses were discussed in a previous review.^[^ ^168^ ^]^ The two key challenges in the synthesis are the stereoselective construction of the cyclohexane core and the installation of the α‐tertiary amine, which forms the anchor of the guanidine group (Scheme 5).

Kishi derived the cyclohexane core from a dearomatization of hydroquinone, while Sato started from *myo*‐inositol, and Fukuyama used benzoquinone in their 2017 synthesis. Instead of using preexisting six‐membered carbocyclic starting materials, ring‐closing reactions were employed by Isobe, Fukuyama‐Yokoshima, as well as Qi through a Diels–Alder reaction, Du Bois through a rhodium‐catalyzed carbene insertion, and Trauner through a 1,3‐dipolar cycloaddition. Although Kishi pursued a racemic synthesis of TTX (**1**), Isobe (levoglucosenone), Du Bois (d‐erythronic acid γ‐lactone), Sato (*myo*‐inositol), Fukuyama–Yokoshima (α‐methyl‐D‐mannoside), and Trauner (d‐glucose) used chiral starting materials to establish an asymmetric synthesis. Fukuyama harnessed an enzymatic lipase‐mediated desymmetrization to separate the racemic starting material, and Qi employed an asymmetric Diels–Alder reaction using (–)‐camphanic acid as chiral auxiliary.

To install the α‐tertiary amine, Kishi used a Beckmann rearrangement, Isobe used an Overman rearrangement, Fukuyama‐Yokoshima used an Ichikawa rearrangement, and Fukuyama used a Hofmann rearrangement. Du Bois employed a rhodium‐catalyzed nitrene insertion, Sato and Qi harnessed a nucleophilic substitution of a chloro epoxide with sodium azide, while Trauner applied a nucleophilic addition of TMS‐acetylene to an oxazoline.

#### 7.1.1 Kishi Synthesis of Tetrodotoxin

**Scheme 6:**
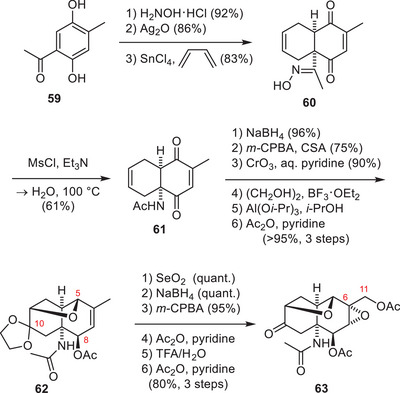
Kishi synthesis of TTX (**1**)–Part 1. Reagents: Ac_2_O = acetic anhydride, mCPBA = *meta*‐chloroperoxybenzoic acid, MsCl = methanesulfonyl chloride, TFA = trifluoroacetic acid.

**Scheme 7:**
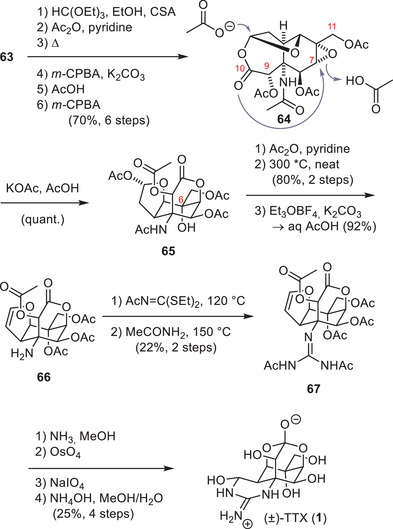
Kishi synthesis of TTX (**1**)–Part 2. Reagents: *m*CPBA = *meta*‐chloroperoxybenzoic acid, CSA = camphorsulfonic acid, TFA = trifluoroacetic acid.

The first synthesis of TTX (**1**) was established by Kishi and coworkers in 1972 (Schemes 6 and 7).^[^ ^9^ ^]^ Their synthesis commenced by the formation of a *cis*‐decalin (**60**) through a SnCl_4_‐mediated Diels–Alder reaction between butadiene and a *para*‐quinone (Scheme 6). The *para*‐quinone was generated through oxime‐formation of **59** with hydroxyamine, followed by oxidation with Ag_2_O.^[^ ^11^, ^169^ ^]^

A Beckmann rearrangement of the ketoxime **60** provided the α‐tertiary amine motif (**61**).^[^ ^11^ ^]^ For the introduction of the TTX (**1**) oxygenation pattern, Kishi and coworkers exploited the effect that reagents/reactants approach from the convex face of the bicyclic *cis*‐decalin structure.^[^ ^9^, ^11^ ^]^ In this context, the C5 stereocenter (**62**) was set through a NaBH_4_ reduction, and the resulting hydroxy group was in an ideal position to open the epoxide that was formed through a Prilezhaev oxidation. The liberated C10 alcohol was oxidized and protected as a dioxolane. A selective Meerwein–Ponndorf–Verley reduction set the C8 hydroxy group, which was acetylated to give **62**. The C11 alcohol (**63**) was introduced by a Riley oxidation–reduction sequence, and the C6 oxygen atom (**63**) was set through a Prilezhaev epoxidation. C10 acetal deprotection was followed by an acetylation procedure and provided **63**.

To install the C9 hydroxy group (**64**, Scheme 7), a Rubottom‐type oxidation was used, which required the formation of an enol ether. This was achieved by the formation of a diethyl ketal from the C10 ketone (**63**) and the thermal extrusion of one ethanol group. Epoxidation of the generated enol ether and hydrolysis with AcOH provided the C9 acetate (**64**).

The C10 ketone was oxidized to the corresponding ester **64** through a Baeyer–Villiger reaction, which set the stage for a cascade cyclization. This cascade was initiated through an attack of an acetate anion onto the acetal moiety (**64**), which liberated the C10 carboxylate. The carboxylate group was ideally positioned to open the epoxide at the C7 position and provided **65** with the desired C7 stereoconfiguration. Acetylation of the C6 alcohol enabled the thermal elimination of the acetate within the tetrahydrofuran ring to give the corresponding dihydrofuran. Cleavage of the amide bond was achieved using Meerwein's salt and gave amine **66,** which was subjected to newly developed guanidylation conditions to afford guanidine **67**.^[^ ^9^ ^]^ To finalize TTX (**1**), the dihydrofuran was oxidatively cleaved, followed by a global acetate deprotection, which provided the natural product in a total of 31 steps from 5‐acetyltoluhydroquinone (**59**).

#### 7.1.2 Isobe Synthesis of Tetrodotoxin

**Scheme 8:**
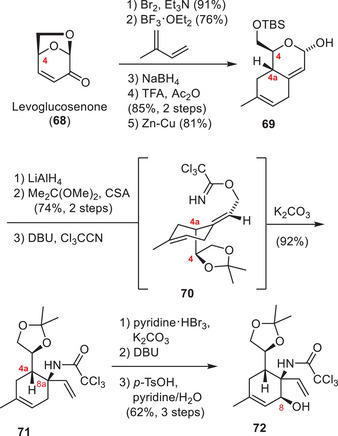
Elaboration of levoglucoseone (**68**) into aminoalcohol **72**. Reagents: TFA = trifluoroacetic acid, DBU = 1,8‐diazabicyclo(5.4.0)undec‐7‐ene, *p*‐TsOH = *para*‐toluenesulfonic acid.

**Scheme 9:**
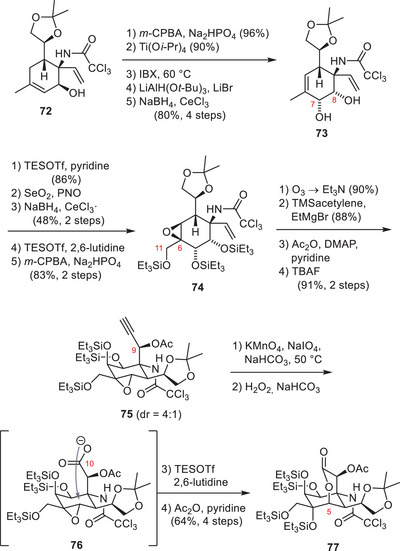
Installation of the TTX (**1**) oxygenation pattern. Reagents: *m*CPBA = *meta*‐chloroperoxybenzoic acid, IBX = 2‐iodoxybenzoic acid.

**Scheme 10:**
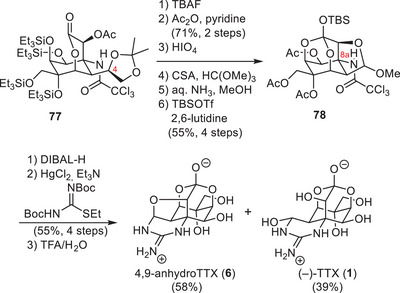
Finalizing TTX (**1**) and 4,9‐anhydroTTX (**6**) from **77**. Reagents: TBAF = tetrabutylammonium fluoride, CSA = camphorsulfonic acid, DIBAL‐H = diisobutylaluminium hydride, TFA = trifluoroacetic acid.

Isobe and coworkers have developed the first asymmetric synthesis of TTX (**1**) in 2003, which furnished the natural product in 77 steps from the chiral starting material 2‐acetoxy glucal.^[^ ^170^, ^171^ ^]^ To increase the utility of their synthesis, they devised an improved version in 2004 starting from levoglucosenone (**68**) (Schemes 8, 9, 10).^[^ ^172^, ^173^ ^]^ As part of their synthesis, the C4 stereocenter of levoglucosenone (**68**) was used to direct a Diels–Alder cycloaddition and provide the six‐membered core carboxycle of TTX (**1**) with the desired C4a configuration (**69**) (Scheme 8).^[^ ^174^ ^]^ Redox and protecting group manipulations provided **70**, which underwent an Overman rearrangement and set the C8a α‐tertiary amine (**71**). Thereafter, the oxygenation pattern of TTX (**1**) was installed on the cyclohexene core (**71**), which could be diastereoselectively directed through the C8a and C4a stereocenters. To install the C8 hydroxy group, the internal alkene (**71**) was dibrominated and subjected to a DBU‐mediated substitution cascade.

This cascade entailed the elimination of the secondary bromide, affording an allylic tertiary bromide. This allylic bromide was displaced by a nucleophilic S_N_2’ attack of the trichloroacetamide oxygen atom.^[^ ^174^, ^175^ ^]^ The resulting oxazoline was hydrolyzed to provide the allylic alcohol **72**. Prilezhaev epoxiation and epoxide‐opening via elimination provided an allylic 1,2‐diol, which was oxidized to the corresponding diketone and subjected to a two‐step reduction sequence to give the desired stereoconfigurations of the C7 and C8 hydroxy groups (**73**, Scheme 9).^[^ ^176^ ^]^

A Riley oxidation‐reduction sequence provided the C11 hydroxy moiety, whereas the C6 oxygen was diastereoselectively installed through a Prilezhaev oxidation, which yielded epoxide **74**. The C9 stereocenter was introduced by ozonolysis of the vinyl group, followed by Cram chelate‐mediated ethynylmagnesium bromide addition into the resulting aldehyde (**75**). Oxidative cleavage of the terminal alkyne using sequential oxidations with KMnO_4_/NaIO_4_ and H_2_O_2_ provided the corresponding C10 carboxylate (**76**), which nucleophilically opened the epoxide and set the C5 stereocenter (**77**).

To finalize TTX (**1**), the 1,2‐diol moiety (**77**, Scheme 10) at C4 was oxidatively cleaved to the corresponding aldehyde, and the C8a α‐tertiary amine (**78**) was transformed into a guanidine using a modified Kishi guanidylation. Global deprotection furnished TTX (**1**) and 4,9‐anydroxTTX (**6**) in 39 steps from levoglucosenone (**68**). Based on their general synthetic approach toward TTX (**1**), Isobe and coworkers have succeeded in synthesizing 11‐deoxyTTX (**10**),^[^ ^176^ ^]^ 5,11‐dideoxyTTX (**13**),^[^ ^175^ ^]^ chiriquitoxin (**32**),^[^ ^177^ ^]^ 5‐deoxyTTX (**12**),^[^ ^178^ ^]^ 5,6,11‐trideoxyTTX (**21**)^[^ ^179^ ^]^ and unnatural 8‐deoxyTTX derivatives.^[^ ^178^ ^]^ Their respective syntheses are discussed in Section 7.2.

#### 7.1.3 Du Bois Synthesis of Tetrodotoxin

**Scheme 11:**
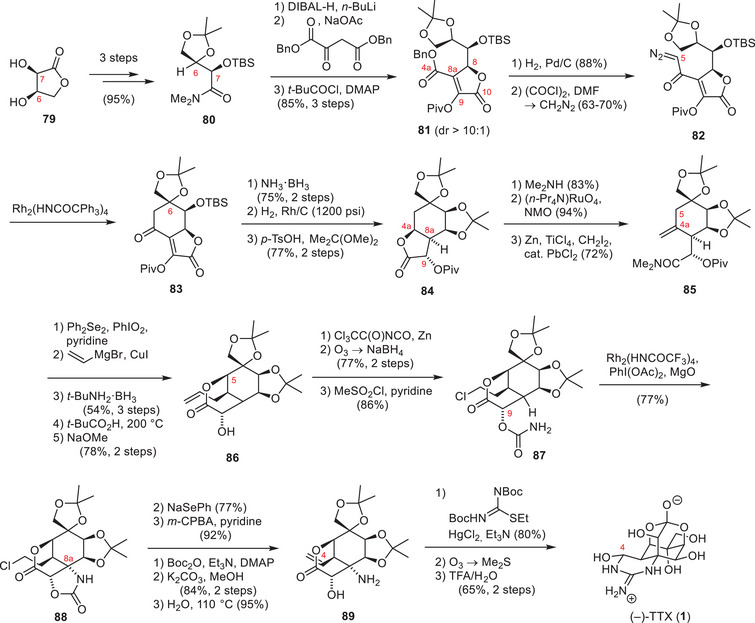
Du Bois synthesis of TTX (**1**). Reagents: DIBAL‐H = diisobutylaluminium hydride, DMAP = 4‐dimethylaminopyridine, *p*‐TsOH = *para*‐toluenesulfonic acid, NMO = *N*‐methylmorpholine *N*‐oxide, *m*CPBA = *meta*‐chloroperoxybenzoic acid.

Du Bois and coworkers have demonstrated the utility of their carbene/nitrene C–H insertion methodologies by establishing an asymmetric synthesis of TTX (**1**) from enantiomerically pure d‐erythronic acid γ‐lactone (**79**, Scheme 11).^[^ ^180^, ^181^ ^]^ d‐Erythronic acid γ‐lactone (**79**) contains two stereocenters that were incorporated into TTX (**1**) as the C6 and C7 hydroxy groups. Therefore, **79** was converted to amide **80** in a three‐step amide formation—protecting group manipulation sequence. An aldol reaction with dibenzyl oxaloacetate was used as a key opening step to set the C8 stereocenter and provide the C9, C10 structural elements as well as the C4a and C8a carbon atoms (**81**). A homologation appended diazomethane (**82**), which was used in the rhodium‐catalyzed carbene C–H insertion to close the cyclohexane core (**83**). Reduction of the C4a carbonyl to the corresponding hydroxy group enabled the subsequent hydrogenation of the alkene, which proceeded from the convex face of the bicycle and set the C8a and C9 stereocenters (**84**). After acetonide protection (**84**), the lactone structure was opened with dimethylamine, and the liberated C4a alcohol was oxidized to a ketone using a Ley oxidation. This C4a ketone was transformed into an exo‐methylene via a Tebbe olefination (**85**), which, in turn, enabled a Riley oxidation of the C5 methylene to a ketone. The exo‐methylene was converted into an allyl group through a metal‐mediated vinyl addition (**86**), and a reduction of the C5 ketone was performed. Acid‐mediated lactone formation onto the resulting C5 alcohol (**86**) set the precursor for the key C–H nitrene insertion. This step relied on the installation of a carbamate group on the C9 alcohol, followed by transformation of the allyl group into an ethyl chloride (**87**). A binuclear rhodium complex catalyzed the carbamate nitrene to insert into the C8a C–H bond and provided an α‐tertiary amine (**88**). The ethyl chloride group was transformed into the C4a vinyl group via a Grieco‐type elimination, and the amine was deprotected using a three‐step sequence (**89**). Kishi guanidylation of the primary amine, ozonolysis of the vinyl group to liberate the C4a aldehyde, and acid‐mediated global deprotection provided TTX (**1**) in a total of 30 steps from d‐erythronic acid γ‐lactone (**79**).

#### 7.1.4 Sato Synthesis of Tetrodotoxin

**Scheme 12:**
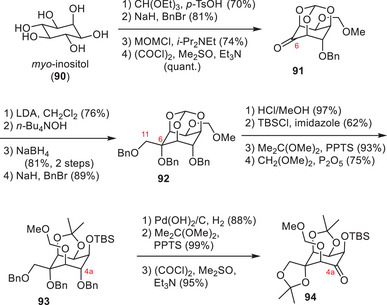
Synthesis of **94** *myo*‐inositol. Reagents: MOMCl = chloromethyl methyl ether, LDA = lithium diisopropylamide, PPTS = pyridinium *para*‐toluenesulfonate.

**Scheme 13:**
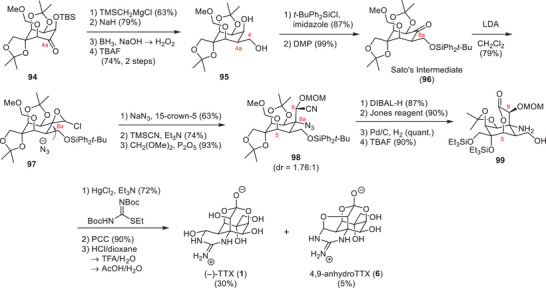
Finalizing TTX (**1**) via Sato's intermediate (**96**). Reagents: DMP = Dess–Martin periodinane, LDA = lithium diisopropylamide, DIBAL‐H = diisobutylaluminium hydride, PCC = pyridinium chlorochromate, TBAF = tetrabutylammonium fluoride.

The approach of Sato and coworkers focused on converting polyhydroxylated sugar derivatives into TTX (**1**).^[^ ^182^, ^183^, ^184^ ^]^ Their first‐generation strategy employed *myo*‐inositol (**90**, Scheme 12) as a chiral starting material and used selective protecting group manipulations to focus the reaction on the desired hydroxy groups.^[^ ^184^ ^]^ The application of an ortho ester, a benzyl, and a MOM group provided a free alcohol at the C6 position, which was oxidized to the ketone **91** using Swern conditions. Installation of a chloro epoxide and base‐mediated epoxide‐opening provided an α‐hydroxy aldehyde, which was reduced to the desired C6, C11 1,2‐diol (**92**). A 6‐step deprotection–protection sequence via **93** afforded the free C4a hydroxy group, which was subjected to a Swern oxidation (**94**). The resulting ketone (**94**, Scheme 13) was converted into an exo‐methylene through a Peterson olefination and further modified to give the primary C4 alcohol via a hydroboration–oxidation procedure (**95**).

Protection of the primary alcohol with *t*‐BuPh_2_SiCl enabled the selective oxidation of the C8a alcohol to the ketone (Sato's intermediate, **96**, Scheme 13), which was reacted with lithium dichloromethide to give the chloro epoxide **97**. Opening of the epoxide (**97**) with sodium azide set the C8a α‐tertiary azide (**98**). A hydrocyanation diastereoselectively (dr = 1.76:1) set the C9 alcohol and introduced the C10 carbon atom. The nitrile **98** was reacted to the corresponding C5‐bonded lactone by DIBAL‐H reduction and Jones oxidation. Hydrogenation of the azide gave the α‐tertiary amine **99** and was followed by Kishi guanidylation as well as the oxidation of the C4 alcohol. Global acid‐mediated deprotection finalized TTX (**1**) in a total of 30 steps from *myo*‐inositol (**90**). Sato and coworkers have established two additional formal syntheses of TTX (**1**), which intercepted their key intermediate **96** and started from d‐glucose.^[^ ^182^, ^183^, ^184^ ^]^

#### 7.1.5 Fukuyama Synthesis of Tetrodotoxin

**Scheme 14:**
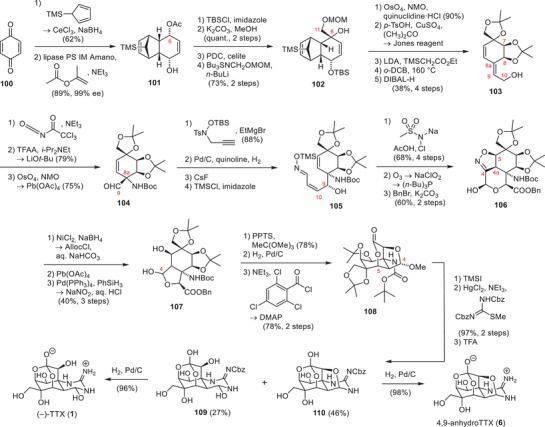
Fukuyama synthesis of TTX (**1**). Reagents: PDC = pyridiniumdichromate, TFAA = trifluoroacetic anhydride, BnBr = benzylbromide, PPTS = pyridinium *para*‐toluenesulfonate, o‐DCB = *ortho*‐dichlorobenzene, DMAP = 4‐dimethylamino pyridine.

In their pursuit of TTX (**1**), Fukuyama and coworkers followed a strategy to form a reversible bicycle and use the bias of reactions to proceed from the convex face of this system to install the oxygenation pattern.^[^ ^185^ ^]^ *Para*‐benzoquinone (**100**, Scheme 14) was reacted in a Diels‐Alder reaction with 5‐TMS‐cyclopenadiene, followed by reduction of the ketones to provide a 1,4‐diol‐substituted *cis*‐fused bicycle (**101**). An enzymatic resolution was used to separate the enantiomers, and the route was continued with the C6 acetate **101**. The C11 hydroxymethyl group (**102**) was diastereoselectively appended through metal‐mediated addition into the corresponding ketone, and the C7, C8 alkene was reacted in an Upjohn dihydroxylation to a 1,2‐diol (**103**). To implement the C9–C10 segment, the C8a alcohol was oxidized and substituted via a Peterson olefination. 5‐TMS‐cyclohexadiene was then thermally extruded to liberate an alkene, and the C10 ester was reduced to the alcohol **103**. The C10 alcohol was substituted with a carbamate unit, which underwent an Ichikawa rearrangement through a reaction with TFAA to give the C8a α‐tertiary amine and a vinyl unit (**104**). Oxidative alkene cleavage afforded the C9 aldehyde (**104**), which was subjected to a metal‐mediated alkynyl addition. The addition followed a Cram chelate transition state and provided the desired stereoconfiguration of the C9 hydroxy group (**105**). The internal alkyne was partially hydrogenation to the *cis*‐alkene, which enabled a 1,3‐dipolar cycloaddition with the cyclohexene unit via the generation of a nitrile oxide from the protected hydroxyamine **105**. This Huisgen cycloaddition resulted in a tricycle and diastereoselectively set the C5 carbon–oxygen bond as well as the C4a carbon–carbon bond (**106**). Oxidation state adjustments on the tricyclic structure were performed through an ozonolysis and a nickel‐catalyzed reduction of the isoxazoline. The resulting 1,2‐aminolactol was oxidatively cleaved, and the amine was extruded from the aminal to provide the C4 lactol (**107**). After a hydrogenative debenzylation liberated the C10 acid, a Yamaguchi esterification gave the six‐membered lactone **108** by coupling with the C5 alcohol. The guanidine was installed using Kishi conditions, which were followed by treatment with TFA to provide a mixture of Cbz‐TTX (**109**) and Cbz‐4,9‐anhydroTTX (**110**). Removal of the Cbz group finalized TTX (**1**) and 4,9‐anhydroTTX (**6**). It is worth noting that Fukuyama and coworkers have additionally synthesized 11‐norTTX‐6(*R*)‐ol (**18**) through a modified version of this route. With this approach, TTX (**1**) could be synthesized from *para*‐benzoquinone (**100**) in a total of 26 steps.

#### 7.1.6 Fukuyama–Yokoshima Synthesis of Tetrodotoxin

**Scheme 15:**
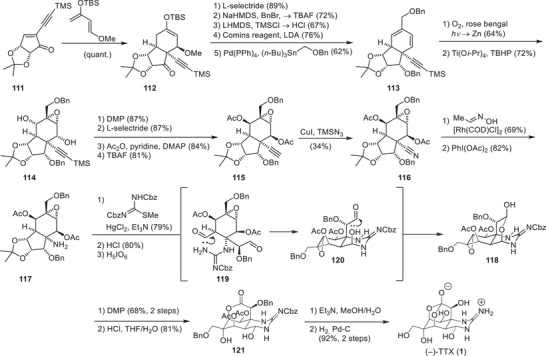
Fukuyama‐Yokoshima synthesis of TTX (**1**). Reagents: NaHMDS = sodium hexamethyldisilazide, LHMDS = lithium hexamethyldisilazide, LDA = lithium diisopropylamide, TBHP = *tert*‐butyl hydroperoxide, DMP = Dess–Martin periodinane, DMAP = 4‐dimethylamino pyridine, TBAF = tetrabutylammonium fluoride.

Fukuyama, Yokoshima, and coworkers continued to pursue their investigations into shorter, more efficient approaches towards TTX by following different synthetic strategies and published a novel approach in 2020 (Scheme 15).^[^ ^186^ ^]^ Here, they exploited the steric bias of a Diels–Alder reaction to construct the C4a and C8a stereogenic centers and introduced the α‐tertiary amine group via a newly developed transformation of a terminal alkyne into a nitrile. The enantiopure dienophile **111** was prepared from α‐methyl‐d‐mannoside according to a literature‐known procedure.^[^ ^187^, ^188^ ^]^ A Diels–Alder reaction with the TBS‐protected version of the Danishefsky diene gave the *cis*‐fused tricycle **112** in quantitative yield. After a diastereoselective reduction of the ketone followed by benzyl protection of the corresponding alcohol, a treatment with TBAF liberated a cyclohexanone structure with the concomitant deprotection of the alkyne. With the reinstallation of the TMS group at the terminal alkyne, the ketone was converted into the corresponding enol triflate using LDA and Comins reagent. A Stille coupling with a benzyl ether introduced the C11 carbon, which contained the benzyl‐protected hydroxy group (**113**). Exploiting the propensity of reagents to proceed from the convex face of a *cis*‐fused bicyclic system, a stereoselective Diels–Alder reaction with singlet oxygen gave an endoperoxide which underwent reductive cleavage with zinc to install the hydroxy moieties at C5 and C8, albeit with the undesired conformation. They harnessed the stereochemical configuration of the two newly formed hydroxy groups in an allylic epoxidation to install the C6 oxygen and yield **114**. A two‐step oxidation‐reduction sequence using Dess–Martin periodinane and l‐selectride afforded the 1,4‐diol with the desired stereochemistry. Acetylation and subsequent TMS deprotection using TBAF gave **115**. The terminal alkyne was next transformed into the sterically demanding α‐tertiary amine group. Treatment of **115** with CuI and TMSN_3_ first formed the nitrile **116** via a Huisgen, retro‐Huisgen reaction cascade, which was followed by a transformation into the corresponding carboxamide using a modified version of Lee´s protocol. A Hofmann rearrangement promoted with PhI(OAc)_2_ gave the free amine **117**. A modified Kishi guanidinylation converted the amine into the *bis*‐Cbz‐protected guanidine. The acetonide was subjected to an acid‐mediated hydrolysis to yield the free diol, which was oxidatively cleaved using periodic acid to initiate a cascade to establish the tricyclic lactol **118**. The free C4 aldehyde on **119** is attacked by the equatorial guanidine, and the resulting axial C4 hydroxy group of the hemiaminal **120** engages the C10 aldehyde to form a lactol **121**. After a Dess–Martin oxidation, the newly formed lactone was hydrolyzed, which led to the epimerization on the C4 stereocenter. The liberated carboxylic acid nucleophilically attacked the epoxide at the C7 carbon, which established the hydroxylated C6 and C7 stereocenters (**121**). Basic removal of the acetyl groups and hydrogenolysis of the benzyl and Cbz protecting groups provided TTX (**1**) in 28 steps from α‐methyl‐d‐mannoside.

#### 7.1.7 Trauner Synthesis of Tetrodotoxin

**Scheme 16:**
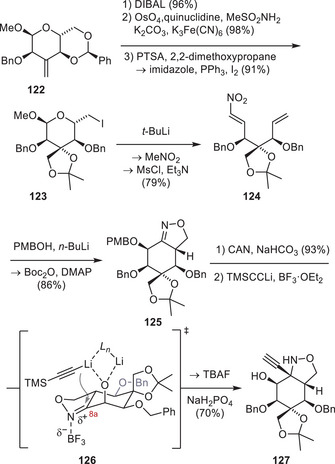
Assembly of the carbon skeleton and installation of the α‐tertiary amine in the Trauner synthesis of TTX (**1**). Reagents: DIBAL = diisobutylaluminium hydride, PTSA = *para*‐toluenesulfonic acid, PMBOH = 4‐methoxybenzyl alcohol, CAN = cerium ammonium nitrate, TBAF = tetrabutylammonium fluoride.

**Scheme 17:**
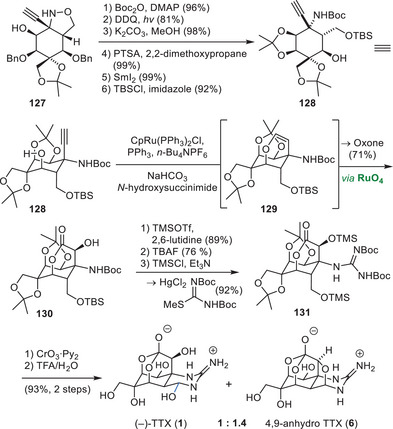
Synthetic endgame sequence of the Trauner synthesis of TTX (**1**). Reagents: DDQ = 2,3‐dichloro‐5,6‐dicyano‐1,4‐benzoquinone, PTSA = *para*‐toluenesulfonic acid, TBAF = tetrabutylammonium fluoride.

To design a concise and high‐yielding synthesis of TTX (**1**), Trauner and coworkers followed a strategy to establish the oxygenation pattern and stereochemistry on a linear precursor, followed by a 1,3‐dipolar cycloaddition to set the cyclohexane core.^[^ ^189^ ^]^ This approach has led to the shortest and highest yielding synthesis to date, with 11% overall yield and 22 steps (Schemes 16 and 17). Specifically, the exo‐methylene **122** was synthesized in three steps on a decagram scale from a commercially available d‐glucose derivate that was originally used by Sato and coworkers in their formal route to TTX (**1**) (Scheme 16). A regioselective reductive cleavage using DIBAL placed a benzyl ether at C5, and a diastereoselective Sharpless dihydroxylation of the exo‐methylene installed the primary alcohol at C11 and the tertiary alcohol at C6 with the desired absolute configuration. Acetonide protection followed by an Appel reaction at C4 gave iodide **123**. Treatment with *t*‐BuLi promoted the reductive Bernet–Vasella fragmentation to yield a δ,ε‐unsaturated aldehyde, which is treated with nitromethane in situ to facilitate a Henry reaction and is followed by a dehydration to give nitro olefin **124**. A diastereoselective oxa‐Michael addition with *p*‐anisyl alcohol and trapping of the intermediate with Boc_2_O triggered the formation of a nitrile oxide and a subsequent 1,3‐dipolar cycloaddition, which afforded isooxazoline **125**. After PMB deprotection using cerium ammonium nitrate (CAN), the addition of lithiated TMS‐acetylidene was directed from the convex face (**126**) of the molecule, and the silyl protecting group was cleaved upon workup to give alkyne **127** as a single diastereomer.

At this stage, all carbons and five out of nine stereocenters with the desired absolute configuration were installed. Following a sequence of protection group manipulations, including a recently developed chromoselective benzyl deprotection and reductive cleavage of the N‐Boc isoxazolidine with SmI_2_, the stage was set for the next key step in their synthesis (Scheme 17). It is worth noting that the DDQ‐mediated debenzylation using green light was a singularly effective and mild procedure that cleaved the benzyl group in the presence of a hydrolytically labile alkyne, an acid‐labile Boc carbamate, and a silyl ether. Alkyne **128** was converted into the bridged dihydropyrane (**129**) with CpRu(PPh_3_)_2_Cl. In situ formation of RuO_4_ with Oxone transformed **129** into the desired hydroxylactone **130** with complete diastereoselectivity by taking advantage of the steric environment provided by the proximal acetonide. Boc removal followed by cleavage of the silyl ethers gave an amino diol, which was in situ protected as *bis*‐trimethyl silyl ether to enable a guanidinylation using Kishi´s conditions to afford **131**. The installation of the C9 and C4 TMS ethers allowed the chemoselective oxidation of the primary C4 alcohol to the corresponding aldehyde, which engaged the guanidine to form a guanidinyl hemiaminal. Treatment with aqueous TFA triggered both the epimerization of C4 and a Boc‐deprotection of the guanidine to give a 1:1.4 mixture of TTX (**1**) and 4,9‐anhydro‐TTX (**6**). This approach has led to the shortest and highest yielding synthesis to date with 11% overall yield and 22 steps from a d‐glucose derivate.

#### 7.1.8 Qi Synthesis of Tetrodotoxin

**Scheme 18:**
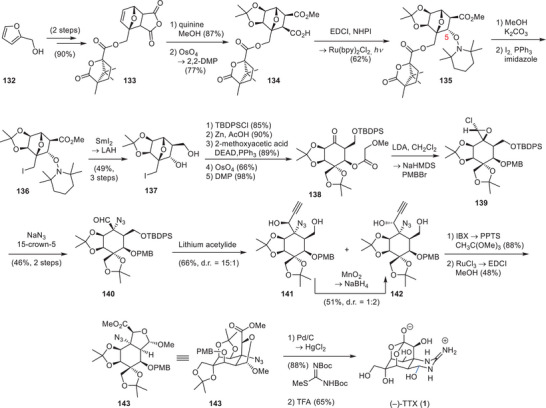
The Qi synthesis towards TTX (**1**) and 9‐*epi*TTX (**16**). Reagents: EDCI = 1‐ethyl‐3‐(3‐dimethylaminopropyl)carbodiimide, NHPI = *N*‐hydroxyphthalimide, DEAD = diethyl azodicarboxylate, DMP = Dess–Martin periodinane, LDA = lithium diisopropylamide, PMBBr = 4‐methoxybenzyl bromide, IBX = 2‐iodoxybenzoic acid, PPTS = pyridinium *p*‐toluenesulfonate, TFA = trifluoroacetic acid.

Many of the previous syntheses of TTX used highly oxygenated starting materials such as sugars and d‐isoascorbic acid with pre‐defined stereochemical conformations. In 2022, the group of Qi and coworkers used a different approach by assembling the highly oxygenated framework early in their synthesis and accessing TTX (**1**) and 9‐*epi‐*TTX (**16**) from a common intermediate (Scheme 18).^[^ ^190^ ^]^ An enantioselective Diels‐Alder reaction of **132** and maleic acid with (–)‐camphanic acid as chiral auxiliar gave **133** as an optically pure compound. Quinine‐mediated regioselective methanolysis, subsequent diastereospecific Upjohn bishydroxylation, and in situ acetalization gave **134**. To install the fifth oxygen moiety at C5, the free acid was transformed into the redox‐active NHPI ester to undergo a Ru‐catalyzed photoredox‐based decarboxylative hydroxylation, which gave **135** as a single diastereomer with inverted configuration at C5. After the removal of the auxiliary through a transesterification, followed by an Appel reaction, provided **136**. Reductive cleavage of the hydroxyamine and simultaneous reduction of the methyl ester gave the free diol **137**. TBDPS protection of the primary alcohol and reductive cleavage of the bridged ether group enabled the stereoinversion of the C5 alcohol via a chemoselective Mitsunobu reaction with 2‐methoxyacetic acid.

These steps were followed by an Upjohn dihydroxylation, in situ protection of the 1,2‐diol with 2‐dimethoxypropane, and Dess–Martin oxidation of the C8a alcohol to provide the ketone **138**. Sato´s dichloromethylation afforded the spiro alpha‐chloroepoxide **139** as a single diastereomer and removed the methoxyesther at C5 to afford the free alcohol, which was subsequently protected with a PMB group. Regioselective opening of the chloroepoxide with sodium azide afforded the azido aldehyde **140**. To construct the dioxaadamantane core and append the guanidinium motif, an addition of lithium acetylide to the aldehyde and removal of the TBDPS group gave the two diastereomeric propargyl alcohols **141** and **142** as a 1:2 mixture, which were used in the divergent synthesis to both TTX (**1**) and 9‐epiTTX (**16**) using the same reaction sequence. It is worth noting that **141** could additionally be recycled to **142** via epimerization using an oxidation‐reduction sequence. IBX oxidation of the primary alcohol and conversion of the newly formed bridged hemiacetal to the corresponding acetal with trimethyl orthoacetate was followed by oxidative cleavage of the alkyne with RuCl_3_/NaIO_4_ and protection of the formed carboxylate as methyl ester to give **143**. Catalytic hydrogenation was employed for both PMB deprotection and azide reduction to the α‐tertiary amine, which was guanidinylated using Kishi's conditions. Global deprotection by treatment with TFA triggered both the formation of the ortho ester and the guanidyl hemiaminal to afford TTX (**1**) in 24 steps starting from furfuryl alcohol **132**. In case **141**, instead of **142**, was subjected to the final four reaction steps, 9‐epiTTX (**16**) was obtained in 22 steps starting from furfuryl alcohol **132**.

### 7.2 Synthesis of Deoxygenated Derivatives and Postulated Biosynthetic Intermediates of Tetrodotoxin

#### 7.2.1 Isobe Syntheses of Deoxygenated Derivatives

**Scheme 19:**
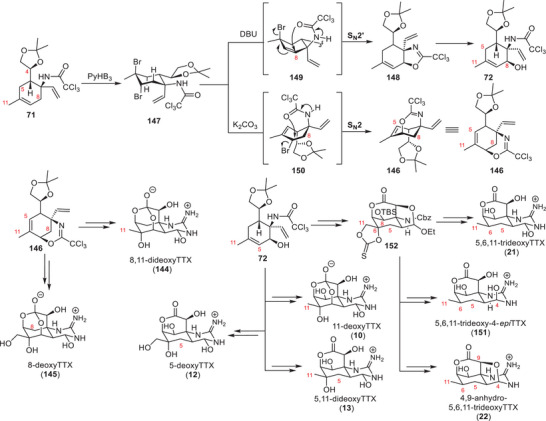
Isobe synthesis of natural and unnatural mono‐, di‐, and trideoxygenated derivatives of TTX (**1**).

Ahead of the publication of their first asymmetric synthesis of TTX, the Isobe group reported the synthesis of various natural products that were isolated from pufferfish.^[^ ^172^, ^175^, ^176^, ^179^, ^191^ ^]^ These compounds share structural features with TTX and are postulated to be biosynthetic precursors. The group used the common retrosynthetic intermediate **71** (Schemes 8 and 19) in their approaches, which allowed them to additionally access unnatural derivatives of TTX (**1**). The first set of molecules that were synthesized included 11‐deoxyTTX (**10**) as well as 5,11‐dideoxyTTX (**13**) and 8,11‐dideoxyTTX (**144**).^[^ ^172^, ^174^, ^176^, ^191^ ^]^ Later, they expanded their approach to yield the 5‐ and 8‐deoxyTTX derivatives **12** and **145**, respectively.^[^ ^178^ ^]^ The derivatives 8,11‐dideoxyTTX (**144**) and 8‐deoxyTTX (**145**) were accessed from the bicyclic imino ether **146**. Their syntheses were guided by the observation that a deoxygenation at C5, C8, and C11 had no significant impact on the assembly of the lactone core and late‐stage guanidinylation.^[^ ^179^ ^]^ Although further deoxygenated analogues on positions 4 and 9 are known (see Section 4), 8‐deoxy derivatives of TTX have not been detected in natural sources to date. It is worth noting that five C8‐*epi* variants were isolated, which were only found in the Japanese sword‐tail newt *Cynops ensicauda popei* in 2019.^[^ ^107^ ^]^ In addition to 8‐*epi*TTX, four derivatives, namely, **24**–**27** (Figure 7), were isolated, which hosted a 5,6,11‐trideoxyTTX substitution pattern.^[^ ^107^ ^]^

Synthetically, all routes relied on the bromination of **71** to **147**, which was instrumentalized in the Isobe route to TTX (**1**) (Scheme 19). Within this intermediate (**147**), only the dioxolane group, which is ultimately transformed into the C4 aldehyde, contributes to the TTX oxygenation pattern. In each synthesis, the introduction of the guanidine moiety was carried out at the latest possible stage due to its observed instability and solubility. Hydroxylation on C11 and C8 was achieved by elaborate oxidation‐reduction sequences and protecting group manipulations, leaving them unaffected for the final steps of the synthesis. Thus, the corresponding deoxy and dideoxy analogues were synthesized using a similar endgame to the one published in their synthesis of TTX (**1**). A central step to install the C8 alcohol entailed the use of a DBU‐mediated formation of the oxazoline ring **148** through a 5‐*exo*‐tet S_N_2’ cyclization of the trichloroacetamide (via the intermediate **149**), which ultimately enabled the synthesis of **72**. To access 8‐deoxytetrodotoxin derivatives, the bicyclic imino ether **146** was formed via a 6‐*exo*‐tet S_N_2 cyclization of the trichloroacetamide (via the intermediate **150**) instead. The natural derivative 5,6,11‐trideoxytetrodotoxin (**21**) was synthesized by using the same route as for the unnatural C4 epimer **151** via the intermediate **152** in 2014.^[^ ^179^ ^]^ Established late‐stage guanidinylation and global deprotection procedures gave a separable mix of 5,6,11‐trideoxyTTX (**21**), 5,6,11‐trideoxy‐4‐epiTTX (**151**), and 4,9‐anhydro‐5,6,11‐trideoxyTTX (**22**).

#### 7.2.2 Ohfune–Shinada Synthesis of 5,6,11‐trideoxyTTX, 4*epi*‐5,6,11‐trideoxyTTX and 4,9‐anhydro‐5,6,11‐trideoxyTTX

**Scheme 20:**
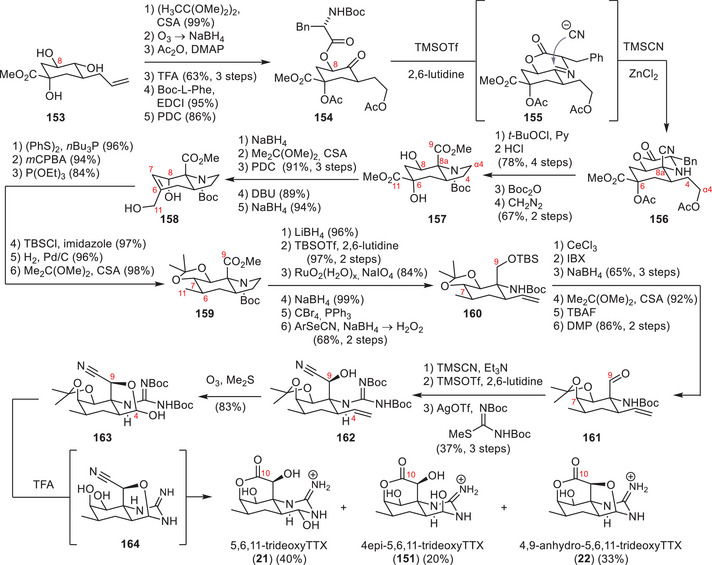
Synthesis of 5,6,11‐trideoxyTTX (**21**), 4*epi*‐5,6,11‐trideoxyTTX (**151**), and 4,9‐anhydro‐5,6,11‐trideoxyTTX (**22**). Reagents: CSA = camphorsulfonic acid, DMAP = 4‐dimethylamino pyridine, TFA = trifluoroacetic acid, EDCI = 1‐ethyl‐3‐(3‐dimethylaminopropyl)carbodiimide, PDC = pyridiniumdichromat, Py = pyridine, DBU = 1,8‐diazabicyclo[5.4.0]undec‐7‐ene, *m*CPBA = *meta*‐chloroperoxybenzoic acid, IBX = 2‐iodoxybenzoic acid, DMP = Dess–Martin periodinane, TBAF = tetrabutylammonium fluoride.

In 2006, Ohfune, Shinada, and coworkers published the synthesis of 5,6,11‐trideoxyTTX (**21**), its C4 epimer (**152**), and 4,9‐anhydro‐5,6,11‐trideoxyTTX (**22**) (Scheme 20).^[^ ^192^ ^]^ The synthesis was guided by the hypothesis that a diastereoselective Strecker reaction would allow for stereospecific construction of the C8a quaternary amino carbon center. Starting from the triol **153**, derived in three steps from quinic acid, a sequence of protections and oxidation state manipulations enabled the regioselective installation of Boc‐l‐Phe ester on the C8 alcohol. Oxidation with PDC gave the α‐ketoester **154**. Mild Boc deprotection using TMSOTf and 2,6‐lutidine formed the cyclic imine **155,** which, upon treatment with TMSCN in the presence of ZnCl, gave the amino nitrile **156**. The Boc‐l‐Phe that served as a chiral auxiliary was removed by oxidation using *tert*‐butyl hypochlorite, which formed the α‐imino nitrile, and was followed by hydrolysis upon refluxing in concentrated hydrochloric acid. HCl treatment additionally led to an acetoxy‐to‐chloride exchange at C4, the hydrolysis of the nitrile group to the carboxylic acid, and the deacetylation of the C6 alcohol. Boc protection of the amine initiated the formation of a pyrrolidine by connecting the C8a amine with the α‐C4 carbon via an S_N_2 reaction, and was followed by an esterification of the C9 carboxylic acid using diazomethane to give the diol **157**. A regioselective reduction of the C11 methyl ester and protection of the resulting C6, C11‐diol gave an acetonide. The C8 alcohol was oxidized to facilitate the elimination of the C6 alcohol through a treatment with DBU and was subsequently reduced back to yield the allylic diol **158**. Introduction of the C7 hydroxy group and simultaneous elimination of the C11 alcohol was achieved by a Mislow–Evans rearrangement. Hydrogenation of the resulting terminal alkene required a prior TBS protection of the allylic alcohol, which was directly transformed into acetonide **159** upon treatment with 2,2‐dimethoxypropane in the presence of CSA. After reduction of the C9 methyl ester and silyl ether protection, the pyrrolidine ring was opened by an oxidation with RuO_4_, followed by reductive cleavage of the resulting lactam using NaBH_4_. An Appel reaction transformed the terminal alcohol into the corresponding bromide, which was subjected to a selenoxide elimination and afforded alkene **160**. Epimerization of the C7 alcohol was achieved by acetonide deprotection using CeCl_3_, followed by IBX oxidation and diastereoselective reduction with NaBH_4_ from the sterically more accessible face of the molecule.

Acetal protection of the resulting diol, followed by a silyl deprotection and Dess–Martin oxidation at C9, provided aldehyde **161**. Treatment of aldehyde **161** with TMSCN and Boc deprotection with TMSOTf of the resulting cyanohydrin enabled the installation of a Boc‐protected guanidine to give **162** as a 1.75:1 mixture in favor of the desired C9 diastereomer. Ozonolysis of the vinyl group of **162** gave the C4 aldehyde, which engaged the C9 alcohol to form lactol **163**. Subjecting **163** to 20% aqueous TFA initiated a global deprotection (**164**) and nitrile hydrolysis to afford 5,6,11‐trideoxyTTX (**21**) together with 4*epi*‐5,6,11‐trideoxyTTX (**151**) and 4,9‐anhydro‐5,6,11‐trideoxyTTX (**22**) as a 10:20:70 mixture.

Treatment with 1% aqueous TFA equilibrated the mixture to yield a 40:26:34 ratio between **21**, **151**, and **22**, which was separated by ion‐exchange column chromatography. Aldehyde **161** was later utilized by the same group to synthesize a ^13^C10 radiolabeled 5,6,11‐trideoxyTTX (**21**), which harnessed Na^13^CN in the presence of MgCl_2_ to increase diastereoselectivity of the cyanohydrin formation, a method developed by Nishikawa et al. in their synthesis of 11‐deoxyTTX (**10**).^[^ ^193^ ^]^

#### 7.2.3 Morimoto Synthesis of the Unnatural 11‐*nor*‐6,7,8‐trideoxyTTX and 11‐nor‐4,9‐anhydro‐6,7,8‐trideoxyTTX

**Scheme 21:**
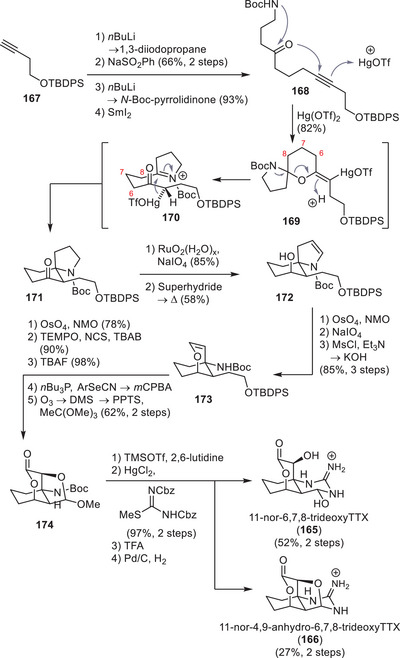
Morimoto synthesis of 11‐*nor*‐6,7,8‐trideoxyTTX (**165**) and 11‐*nor*‐4,9‐anhydro‐6,7,8‐trideoxyTTX (**166**). Reagents: NMO = *N*‐methylmorpholin‐*N*‐oxid, TEMPO = 2,2,6,6‐tetramethylpiperidinyloxyl, NCS = *N*‐chlorosuccimide, TBAB = tetrabutylammonium bromide. *m*CPBA = *meta*‐chloroperoxybenzoic acid, DMS = dimethylsulfide, TFA = trifluoroacetic acid.

In their pursuit to develop new synthetic strategies to construct the cyclohexane framework of TTX derivatives, Morimoto and coworkers published the synthesis of the unnatural 11‐*nor*‐6,7,8‐trideoxyTTX (**165**) and 11‐*nor*‐4,9‐anhydro‐6,7,8‐trideoxyTTX (**166**) (Scheme 21).^[^ ^194^ ^]^ Their synthesis started with the alkylation of but‐3‐yn‐1‐ol silyl ether (**167**) using 1,3‐diiodopropane. Subsequent sulforylation of the terminal iodide enabled the α‐deprotonation followed by acylation with *N*‐Boc‐pyrrolidinone. Desulfonylation using SmI_2_ gave **168** as precursor for their key cyclization step. Treatment with catalytic amounts of mercury triflate triggered a 6‐*exo*‐dig intramolecular oxymercuration by activating the alkyne π‐system. It was hypothesized that a nucleophilic attack of the protected amine gives the spirocyclic aminal **169** as an intermediate. Acidic cleavage leads to iminium **170**, which can undergo a Petasis–Ferrier‐type cyclization to provide the spirocyclic amine (**171**) while regenerating the catalyst. Oxidation of the pyrrolidine to the lactam using RuO_4_, followed by global reduction using superhydride, gave a diol which dehydrated upon refluxing in HMPA to give pyrroline **172**. Lemieux–Johnson oxidation led to the formation of a lactol with the C7 alcohol and was followed by an elimination with MsCl and Et_3_N to give vinyl ether **173**. Dihydroxylation with OsO_4_ and NMO is followed by a chemoselective oxidation of the lactol group to the α‐hydroxylactone. It is worth noting that the following deprotection of the silyl ether with TBAF didn´t give the desired product with a shorter chain length. This required the removal of a terminal C1 unit, which was achieved by a Grieco elimination protocol followed by ozonolysis of the terminal alkene to the corresponding aldehyde and acetal protection of the spontaneously formed hemiacetal to give tricyclic **174**. *N‐*Boc deprotection with TMSOTf in the presence of 2,6‐lutidine, guanidinylation, and global deprotection afforded 11‐*nor*‐6,7,8‐TTX (**165**) and 11‐*nor*‐4,9‐anhydro‐6,7,8‐TTX (**166**).

#### 7.2.4 Nishikawa Synthesis of the Postulated Maritime Biosynthetic TTX Intermediates Tb‐210B, Tb‐226, Tb‐242C, and Tb‐258

**Scheme 22:**
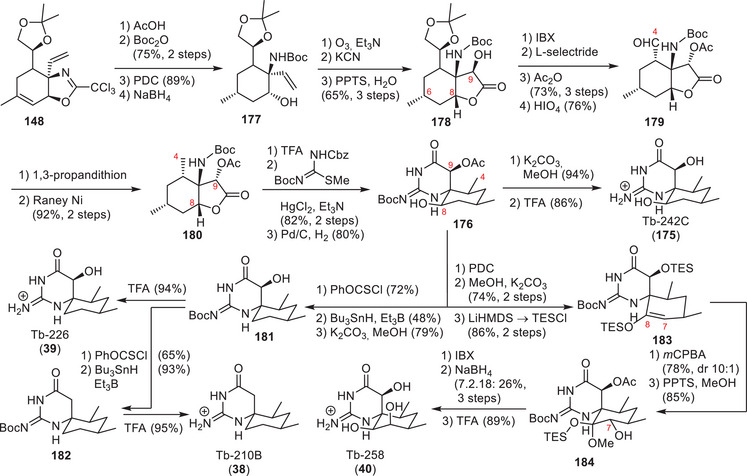
Synthesis of the proposed early TTX precursors Tb‐210B (**38**), Tb‐226 (**39**), Tb‐242C (**175**), and Tb‐258 (**40**) in pufferfish. Reagents: PDC = pyridiniumdichromate, PPTS = pyridinium *p*‐toluenesulfonate, IBX = 2‐iodoxybenzoic acid, LiHMDS = lithium bis(trimethylsilyl)amide, *m*CPBA = *meta*‐chloroperoxybenzoic acid, TFA = trifluoroacetic acid.

In 2022, Toshio Nishikawa, who worked on the first asymmetric TTX synthesis with Minoru Isobe twenty years prior, published the synthesis of the spirocyclic guanidines Tb‐210B (**38**), Tb‐226 (**39**), Tb‐242C (**175**), and Tb‐258 (**40**).^[^ ^195^ ^]^ As discussed in Section 5, they are proposed biosynthetic precursors of TTX in marine species. The strategy of the Nishikawa group entailed accessing the *spiro*‐guanidine **176** as a common intermediate, which yields the target compounds by a stepwise manipulation of the oxidation state (Scheme 22). From the known oxazoline **148**, the dimethylcyclohexane core was stereoselectively assembled. After hydrolysis of the heterocycle and Boc protection of the α‐tertiary amine, the allylic alcohol was oxidized with PDC. A conjugate reduction gave alcohol **177** as the desired epimer on C8 and C6. Ozonolysis and the addition of potassium cyanide, followed by the hydrolysis of the cyclic imino ether, provided lactone **178**. Inversion of C9‐OH was achieved by oxidation with IBX and reduction using l‐selectride. Subsequent protection as acetate ester and deprotection of the acetonide using periodic acid gave the free aldehyde **179**. Deoxygenation on C4 was achieved via the formation of a 1,3‐dithiolane with 1,3‐propandithion followed by desulfurization with Raney nickel to give lactone **180**. Treatment with TFA and a Kishi‐type guanylation using Boc‐ and Cbz‐protected isothiourea formed a guanidine which, upon hydrogenolytic deprotection, prompted the spontaneous cyclization to give the *spiro*‐guanidine **176**. Tb‐210B (**38**), Tb‐226 (**39**), and Tb‐242C (**175**) were collectively accessed by a coordinated sequence of deprotection and targeted Barton–McComby deoxygenations (via **181** and **182**).^[^ ^195^ ^]^ Tb‐258 (**40**) was synthesized by installing the C7 hydroxy group via a Rubottom oxidation: PDC oxidation and saponification of the acetate ester, followed by treatment with LiHMDS and TES chloride, gave the *bis* silylether **183**. Oxidation with *m*CPBA and selective silyl deprotection yielded **184**, bearing the undesired conformation at the C7 position. Epimerization via IBX oxidation, followed by reduction with sodium borohydride and global deprotection using TFA, gave the target compound Tb‐258 (**40**).

#### 7.2.5 Nishikawa Synthesis of the Postulated Terrestrial Biosynthetic TTX Precursors Cep‐210 and Cep‐212

**Scheme 23:**
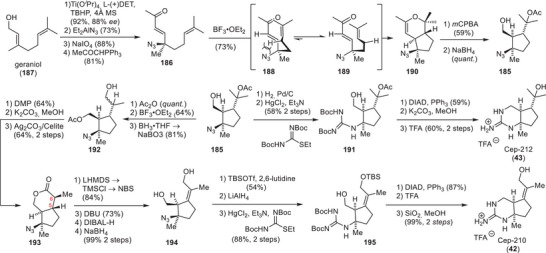
Synthesis of the hypothesized terrestrial tetrodotoxin precursors Cep‐210 (**42**) and Cep‐212 (**43**). Reagents: l‐(+)‐DEP = (+)‐diethyl l‐tartrate, TBHP = *tert*‐butyl hydroperoxide, *m*CPBA = *meta*‐chloroperoxybenzoic acid, DMP = Dess–Martin periodinane, DIAD = diisopropyl azodicarboxylate, DBU = 1,8‐diazabicyclo[5.4.0]undec‐7‐ene, DIBAL‐H = diisobutylaluminium hydride.

In 2019, Nishikawa and coworkers published the asymmetric synthesis of the proposed biosynthetic precursors of TTX in terrestrial species: Cep‐210 (**42**) and Cep‐212 (**43**), isolated from the Japanese toxic newt ***C*** *ynops* ***e*** *nsicauda* ***p*** *opei* (Scheme 23). Both compounds were synthesized from the common intermediate **185**, which was accessed through an intramolecular hetero Diels‐Alder reaction (IHDA) from **186**. A Sharpless asymmetric epoxidation with l‐(+)‐DET of geraniol (**187**) introduced an epoxide in an enantioselective manner, which was regioselectively opened with diethylaluminium azide. Periodate cleavage followed by a Wittig reaction gave enone **186**. Treatment with boron trifluoride etherate in the presence of mol sieves triggered a diastereoselective IHDA. The selectivity was guided by a 1,3‐allylic strain between the enone and the adjacent functional groups, which favors the *exo* transition state **188** over the *endo* transition state **189** and yields enol ether **190**. Oxidative cleavage using *m*CPBA, followed by reduction using NaBH_4_, provided azido alcohol **185** as a common intermediate for both natural products. Azide reduction followed by Kishi guanidinylation gave guanidine **191**. Mitsunobu conditions furnished a heterocyclic six‐membered ring that incorporates the guanidine. Global deprotection using potassium carbonate in methanol, followed by treatment with TFA, provided Cep‐212 (**43**).

The challenge in the synthesis of Cep‐210 (**42**) was the implementation of the olefin geometry of the allyl alcohol, which was achieved through an intermediate lactone formation: A sequence of acetylation, regioselective acetate elimination, and hydroboration–oxidation provided the alcohol **192**. Dess–Martin oxidation, deacetylation, and Fetizon oxidation of the lactol gave the corresponding lactone **193**. The conformation of the C5─C6 bond within the lactone builds the foundation for the formation of the desired allyl alcohol isomer, which is installed through a bromination‐elimination sequence followed by two reductions using DIBAL‐H and NaBH_4_, respectively, to give **194**. Selective protection of the allylic alcohol followed by azide reduction and guanidinylation formed **195**, which was transformed into the natural product through a cyclization via Mitsunobu reaction and sequential global deprotection using TFA, followed by treatment with silica gel in methanol.

It is worth mentioning that the group performed an asymmetric synthesis of Cep‐210 (**42**) and Cep‐212 (**43**) to determine the absolute configuration of the isolated natural products. However, they could not achieve a level of separation for the two enantiomers via chiral HPLC chromatography necessary for an unambiguous assignment. The chiral HPLC separation could be optimized to finally determine the absolute configuration by derivatizing the isolated and synthesized natural products using dinitrofluorobenzene. The stereochemistry of both Cep‐210 (**42**) and Cep‐212 (**43**) was found to be in accordance with TTX (**1**).

## 8 Conclusion

In conclusion, we have provided a detailed account of the research that is targeted to the tetrodotoxin natural product family, with the main focus on tetrodotoxin itself. Our discussion included the mechanism of VGSC blockage, the variety of natural products within this class, and their origins, as well as their bioactivities, biosynthetic hypotheses, and the reported chemical syntheses.

## Supporting Information

A list of bioactivity data for tetrodotoxin and its derivatives relevant for this review is added to the manuscript in the form of a Supporting Information, where the authors have cited additional references.

## Conflict of Interests

M.M. and C.D. are employees of Grünenthal GmbH.

## Supporting information



Supporting Information

## Data Availability

The data that support the findings of this study are available in the supplementary material and the references of this article.
